# Resequencing of 297 melon accessions reveals the genomic history of improvement and loci related to fruit traits in melon

**DOI:** 10.1111/pbi.13434

**Published:** 2020-06-30

**Authors:** Shi Liu, Peng Gao, Qianglong Zhu, Zicheng Zhu, Hongyu Liu, Xuezheng Wang, Yiqun Weng, Meiling Gao, Feishi Luan

**Affiliations:** ^1^ Key Laboratory of Biology and Genetic Improvement of Horticulture Crops (Northeast Region) Ministry of Agriculture and Rural Affairs Northeast Agricultural University Harbin City Heilongjiang Province China; ^2^ College of Horticulture and Landscape Architecture Northeast Agricultural University Harbin City Heilongjiang Province China; ^3^ USDA‐ARS Vegetable Crops Research Unit Horticulture Department University of Wisconsin Madison City WI USA; ^4^ College of Life Sciences, Agriculture and Forestry Qiqihar University Qiqihar City Heilongjiang Province China

**Keywords:** *Cucumis melo*, selective sweep, domestication, improvement, population structure, comparative genomics

## Abstract

Domestication and improvement are two important stages in crop evolution. Melon (*Cucumis melo* L.) is an important vegetable crop with wide phenotypic diversity in many horticultural traits, especially fruit size, flesh thickness and aroma, which are likely the results of long‐term extensive selection during its evolution. However, selective signals in domestication and improvement stages for these remarkable variations remain unclear. We resequenced 297 wild, landrace and improved melon accessions and obtained 2 045 412 high‐quality SNPs. Population structure and genetic diversity analyses revealed independent and two‐step selections in two subspecies of melon: ssp. *melo* and ssp. *agrestis* during melon breeding. We detected 233 (~18.35 Mbp) and 159 (~17.71 Mbp) novel potential selective signals during the improvement stage in ssp. *agrestis* and spp. *melo*, respectively. Two *alcohol acyltransferase* genes (*CmAAT*s) unique to the melon genome compared with other cucurbit crops may have undergone stronger selection in ssp. *agrestis* for the characteristic aroma as compared with other cucurbits. Genome‐wide association analysis identified eight fruit size and seven flesh thickness signals overlapping with selective sweeps. Compared with thin‐skinned ssp. *agrestis*, thick‐skinned ssp. *melo* has undergone a stronger selection for thicker flesh. In most melon accessions, *CmCLV3* has pleiotropic effects on carpel number and fruit shape. Findings from this study provide novel insights into melon crop evolution, and new tools to advance melon breeding.

## Introduction

Crop cultivation started from domestication of wild plants resulting in morphological and physiological changes that distinguish domesticated crops from their wild relatives (Hancock, 2005; Harlan, 1992). The initial domestication was often followed by a process of crop improvement or breeding through diversifying selection, which directed more attention to traits such as yield, colour, flavour and physiological traits contributing to uniformity (Doebley *et al*., 2006; Meyer and Purugganan, 2013). This may be the result of positive or negative selection by farmers during millennia (Pitrat, 2013). Understanding the genetic basis of domestication‐related traits may provide insights into crop evolution. In the last decade, next‐generation sequencing technologies have provided a powerful tool to elucidate the ‘stories of plant breeding history’ in a number of crops such as grape (Myles *et al*., 2011), maize (Hufford *et al*., 2012), cucumber (Qi *et al*., 2013), tomato (Lin *et al*., 2014), soya bean (Zhou *et al*., 2015), rice (Meyer *et al*., 2016), *Brassica rapa* and *Brassica oleracea* (Cheng *et al*., 2016), peach (Li *et al*., 2019), watermelon (Guo *et al*., 2019), melon (Zhao *et al*., 2019) and wax gourd (Xie *et al*., 2019). These studies have revealed that modern cultivated accessions diverged from their wild progenitors in response to human selection fostering interdependence between human and plants.

Melon has been cultivated for at least 4000 years (Pitrat, 2016), which is evidenced from archaeological records in the Lower Egypt (El Hadidi *et al*., 1996; van der Knaap *et al*., 2014), China (Luan *et al*., 2008), Indus valley (Vishnu‐Mittre, 1974) and Iran (Costantini, 1977) that date back to 2000 BCE. Africa, Asia (Dwivedi *et al*., 2010) and Australian (Sebastian *et al*., 2010) were thought to be the three potential regions of melon origin. Endl and colleagues proposed that melon was domesticated at least twice in Africa and Asia (Endl *et al*., 2018). Zhao reported three independent domestication events during melon evolution, two in India and one in Africa (Zhao *et al*., 2019). Melon was initially classified into two subspecies (*C*.* melo* ssp.*melo* and ssp.*agrestis*) based on the presence of ovary hairs (Jeffrey, 1980; Kirkbride, 1993). The ssp.*agrestis* is distributed mainly in Africa, Asia and Australia with *momordica*, *acidulus*, *conomom*, *makuwa* and *chinensis* groups, in Africa with the *tibish* group and in Central America with the *chito* group. The ssp.*melo* including the *cantalupensis*, *inodorus*, *chandalak*, *ameri*, *flexuosus*, *chate* or *dudaim* groups is distributed mainly in India, central and western Asia, Africa, Europe and Americas (Pitrat, 2013 and Pitrat, 2016). Compared with ssp. *agrestis*, ssp.*melo* plants in general exhibit more vigorous vegetative growth, and the fruits have thicker flesh, higher sugar content, and higher biotic and abiotic stress tolerances. Based on the level of domestication or breeding, melon collections could also be recognized into wild types, landraces and improved varieties (Pitrat, 2016).

Since the release of the melon draft genome (Garcia‐Mas *et al*., 2012), a number of domestication‐related or diversifying selection‐related genes or quantitative trait loci (QTLs) have been cloned or characterized (e.g. Argyris *et al*., 2017; Cohen *et al*., 2014; Diaz *et al*., 2017; Feder *et al*., 2015; Tzuri *et al*., 2015). The population structure, selective signals and genome‐wide association analysis were also investigated to understand melon evolution (Esteras *et al*., 2013; Gur *et al*., 2017; Leida *et al*., 2015; Nimmakayala *et al*., 2016; Pavan *et al*., 2017; Tomason *et al*., 2013; Zhao *et al*., 2019). However, in general, the selective signals for the ‘two‐step’ evolution (from wild type to landrace and then to improved varieties) were not well‐understood, especially from the landraces to the improved cultivars. In the present study, we conducted whole‐genome resequencing of 297 melon accessions with significant portion of ssp. a*grestis* accessions that were underrepresented in previous studies. Selective sweeps and novel GWAS signals associated with fruit size, flesh thickness and aroma accumulation during melon domestication and improvement were identified. These findings will increase our understanding of melon domestication, which also provide a powerful tool for molecular breeding.

## Results

### Genomic variants and phylogenetic relationships of 270 melon accessions

**Figure 1:**
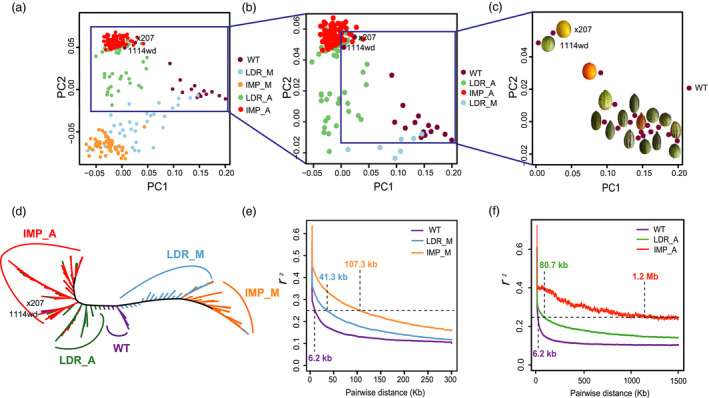
Phylogenetic relationships and linkage disequilibrium of 270 melon accessions. a‐c, PCA plots of the first two components of melon populations. Representative wild melon fruits are shown in (c). d, Phylogenetic tree of the melon populations constructed using 18 510 SNPs at fourfold degenerate sites of melon accessions. e, Genome‐wide average LD decay estimated from WT, LDR_M and IMP_M groups. f, Genome‐wide average LD decay estimated from WT, LDR_A and IMP_A groups. WT, wild type; LDR_A/_M, landraces of ssp. *agrestis/* ssp. *melo*; IMP_A/_M, improved varieties of ssp. *agrestis/* ssp. *melo*.

**Figure 2:**
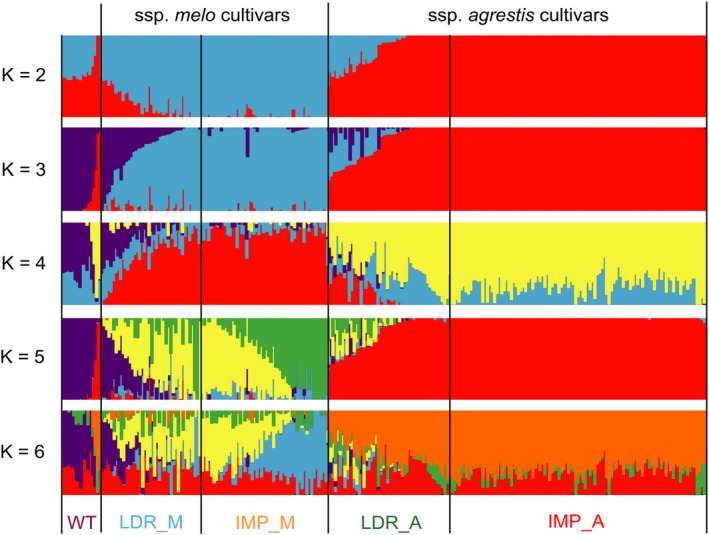
Population structure of 270 melon accessions. *K* = 2, 3, 4, 5 and 6, the y‐axis quantifies subgroups membership, and the *x*‐axis lists the different accessions. The orders and positions of these accessions on the *x*‐axis are the same as the first 270 lines in Table S1.

We resequenced 297 accessions, but only 270 had unambiguous information on their taxonomic status (subspecies: ssp. *melo* or ssp. *agrestis*), or improved status (landrace, improved or wild type) details in Table S1. Thus, for accurate inference, only the 270 accessions were sued in phylogenomic analysis, while all 297 lines were included in GWAS analysis below. In total, 735 Gb resequencing data for the 297 accessions were generated with an average of ~5× depth coverage, from which, 2 045 412 high‐quality SNPs were called against the melon reference genome (V3.5.1 version). The subgroup division was evident from the maximum‐likelihood (ML) phylogenetic tree with 18 510 4DTv SNPs (Figure 1). Among the 16 WT (wild type) accessions, 11 were clustered in one clade, 3 were scattered near the LDR_A (landrace ssp. *agrestis*) clade, and the remaining 2 ‘Mapao’ melons (‘x207’ and ‘1114wd’, Table S1, similar with wild melon morphologically) were located within IMP_A (improved ssp. *agrestis*). The domesticated melon accessions were grouped into two major clades designated as ssp. *agrestis* and ssp. *melo*. Each major clade included both landraces and improved cultivars. The two improved subgroups, improved ssp. *melo* (IMP_M) and improved ssp. *agrestis* (IMP_A), were clearly differentiated from their landrace ssp. *melo* (LDR_M) and landrace ssp. *agrestis* (LDR_A), respectively. The principal component analysis (PCA) provided similar results as the two subspecies were well‐separated from each other (Figure 1a to c). The nucleotide diversity (π) of the WT group was 1.18 × 10^−3^, which was 0.98 × 10^−3^ in LDR_A and 0.28 × 10^−3^ in IMP_A suggesting significant reduction in the genetic diversity during the improvement of IMP_A from LDR_A. In ssp. *melo* accessions, the π value was 1.70 × 10^−3^ in LDR_M and 1.30 × 10^−3^ in IMP_M. The two subspecies were thought to be domesticated from different WT pools, and the nucleotide diversity of WT ssp. *melo* was higher than WT ssp. *agrestis* (Zhao *et al*., 2019). The LD varied significantly among the 5 subgroups (Figure 1e and f), and the LD decay distance (to *r* ^2^ = 0.25) for LDR_A, IMP_M, LDR_M and WT was 80.7, 107.3, 41.3 and 6.2 kb, respectively. For IMP_A, the LD decay distance was approximately 1.2 Mb (to *r* ^2^ = 0.25). The much lower π value and longer LD distance for the IMP_A group suggested that this group may have undergone a severe bottleneck. We ran ADMIXTURE 1.3.0 software with *K* values from 2 to 6. At *K* = 2, except WT group, cultivars (include landraces and improved varieties) of ssp. *melo* and *agrestis* were formed. Five subgroups could be recognized at *K* = 3 (Figure 2). Of the 16 WT accessions, 8 had a uniform and consistent ancestry, whereas the remaining 8 contained varying degree of ancestries from the LDR_A clade (Figure S1). Most accessions in LDR_M and LDR_A carried different levels of WT ancestries indicating varying degrees of domestication. Nearly all the individuals in IMP_M and IMP_A were uniform with trace ancestry suggesting high degree of selection during the improvement from landraces. Taken together, these results supported the recent finding that ssp. *agrestis* and ssp. *melo* represented the two independent domestication events during melon evolution (Zhao *et al*., 2019). Based on results from our work, the two Mapao melon accessions were excluded from the WT subgroup in all subsequent analyses.

### Selective sweeps during melon evolution

We identified 207 and 217 potential XP‐CLR selective signals (Table S2) of WT versus LDR_A and WT versus LDR_M (Figure S2) covering 20.15 Mb (~5.30%) and 18.77 Mb (~4.94%) of the assembled melon genome, respectively. Meanwhile, 258 and 186 potential selective signals accounting for ~19.78 Mb and 20.22 Mb genome (Table S3) were detected in LDR_A versus IMP_A and LDR_M versus IMP_M. Approximately 10.60 Mb selective chromosome regions overlapped between WT versus LDR_M and WT versus LDR_A, occupying ~52.61% and ~56.74% of the total top 5% XP‐CLR regions in each comparison. However, the overlapped sweeps were dropped to 1.75 Mb (only occupying ~8.26% and ~8.85% of the total top 5% of XP‐CLR regions in each comparison) in the improved stage. In the improvement sweeps for ssp. *agrestis* and ssp. *Melo,* 1160 and 1283 genes were annotated, respectively (Tables S4 and S5). Among them, only 61 shared between the two subspecies (Table S6) suggesting differential selections in the two taxa during melon breeding. We aligned the detected selective sweeps in the improvement stage to those reported melon putative domestication sweeps by Zhao *et al*. (2019), and 25 (~1.43 Mb) and 27 (~2.51 Mb) sweeps were consistent on chromosome locations for ssp. *agrestis* and *melo*, respectively, suggesting novel selected regions detected during the improvement stage by our work. *F*_ST_ values were also estimated (Tables S7 and S8), and regions under both top 5% XP‐CLR and top 5% *F*_ST_ were considered as the putative selective sweeps for further analysis (Table S9). Comparisons of XP‐CLR and *F*_ST_ values between the LDR_M and LDR_A, as well as between IMP_M and IMP_A, showed that most of the SNPs contained a high XP‐CLR and *F*_ST_ values, especially for the IMP_M versus IMP_A comparison, suggesting that selection during improvement has resulted in quite different genetic makeups in the two subspecies. The obvious differences in XP‐CLR and *F*_ST_ values for the two subspecies suggested that ssp. *melo* and ssp. *agrestis* have undergone differential selections during evolution and especially in improvement selection.

XP‐CLR and *F*_ST_ analyses revealed a region harbouring 5 genes annotated as *CmAATs* (*MELO3C024762*, *MELO3C024764*, *MELO3C024766*, *MELO3C024769* and *MELO3C024771;* Figure S3a to e) that seemed to be under intensive selection. The 5 *CmAATs* clustered in a ~251.3‐kb region on chromosome 11 with top 2.75% XP‐CLR and top 0.24% *F*_ST_ values between WT and LDR_A. The π and Tajima’s *D* values of the 251.3 kb target region in WT were higher than those in LDR_A. We examined expression patterns of the 5 *CmAATs* during melon development using public RNA‐seq data and found 3 *CmAATs* (*MELO3C024766*, *MELO3C024769* and *MELO3C024771*) with their expression correlated with fruit development. *MELO3C024766* and *MELO3C024771* increase expression level sharply around 30 days after anthesis (DAA), which peaked at 40–45 DAA in matured melon flesh and pericarp (Figure S4a to c and i to k). In contrast, the expression in flower, root and leaf was notably low at the mature stage (Figure S4d and l). The expression of *MELO3C024769* was peaked at 30 DAA in flesh and decreased rapidly in the flesh of mature fruit (Figure S4e and f), while at the mature stage, it reached to the peak in fruit rind (Figure S4g). *MELO3C024769* expression was quite low compared with *MELO3C024766* and *MELO3C024771* throughout the development stages. Totally, 10 SNPs were identified in exonic regions of the 3 *CmAATs* (*MELO3C024766*, *MELO3C024769* and *MELO3C024771*) between wild types and domesticated accessions (both landraces and improved cultivars), 5 of which caused amino acid substitutions in *MELO3C024766* (*S11_7918549*, *S11_7919169*) and *MELO3C024771* (*S11_8043933*, *S11_8044338* and *S11_8044448*). We also detected the variations of these 5 SNPs with our GAWAS panel. Nearly all the 50 LDR_A accessions contained the 5 nonsynonymous SNPs compared with WT accessions. The mature fruit of most LDR_A, but none of the WT accessions, accumulated aroma in the fruit skin and/or flesh. However, the same nonsynonymous SNPs were also identified in most accessions of LDR_M, IMP_M and IMP_A. In *MELO3C024771*, *S11_8043933* exhibited a higher correlation between wild types and domesticated cultivars than *S11_8044338* and *S11_8044448* (Table S10). Two wild melon (PI 614575 and PI 614574) and one landrace (PI 200817) carried the same haplotypes of *S11_8044338* and *S11_8044448* as modern varieties, but did not generated any aroma. This implies that the haplotype of *S11_8043933* was more important in *MELO3C024771* (*CmAAT1*, Galaz *et al*., 2013). *S11_7918549* in *MELO3C024766* (*CmAAT2*, Galaz *et al*., 2013) exhibited a higher correlation than *S11_7919169* among aroma‐producing domesticated melon accession. We also found that nearly all the aromatic melon accessions contained the domesticated haplotype of *S11_8043933* and *S11_7919169* in *CmAAT1* and *CmAAT2*, respectively. However, some domesticated accessions, such as x091 and m4‐75 (*C*.* melo* ssp.*flexuosus*) still carried the same alleles in *MELO3C024771* and *MELO3C024766* as the wild type melons that had no aroma through fruit development.

We also examined the roles of the 5 *CmAAT* homologous genes in other cucurbits from publicly RNA‐seq data. Homologs of *MELO3C024762*, *MELO3C024764* and *MELO3C024766* have been identified in cucumber, watermelon, bottle gourd and pumpkin/squash. Expression of the cucumber gene *Csa2G429040* (homolog of *MELO3C024766*) increased with fruit development in both the flesh and peel, which peaked at mature fruit (Figure S5a). *CmoCh20G009650* was also a homolog of *MELO3C024766* in pumpkin, which expressed only in mature fruit but at a relatively low level (Figure S5b), whereas homologous genes *MELO3C024762* and *MELO3C024764* in other cucurbits did not seem to be associated with fruit development. No homologs of *MELO3C024769* and *MELO3C024771* were detected in other cucurbits, which are probably unique to the melon genome because of their critical roles in aroma generation.

### Candidate selective sweeps for fruit size

**Figure 3:**
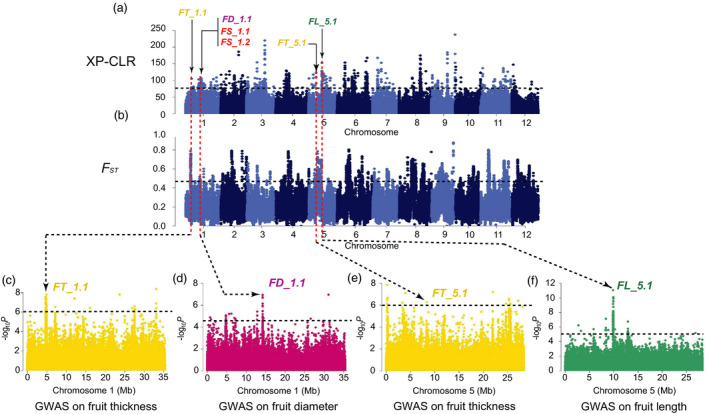
Independent selections on fruit size and flesh thickness in ssp. *agrestis* during domestication. a, Selective signals of ssp. *agrestis* in domestication stage from wild melons to landraces. b, Genome‐wide *F*_ST_ estimates from wild melon to landrace of ssp. *agrestis*. Black horizontal dashed lines indicate the genome‐wide threshold of selection signals and population differentiation index. Red horizontal dashed lines in a and b indicate the same locations between the potential selective sweeps and the population differentiation. Previously reported QTL for fruit size (red), fruit length (FL, green), fruit diameter (FD, dark pink) or flesh thickness (FT, yellow), and GWAS signals identified in this study overlapped with selective sweeps are marked. c to f, Manhattan plots of GWAS for fruit length (f), fruit diameter (d) and flesh thickness (c, e). *FT_1.1*, *FD_1.1*, *FT_5.1* and *FL_5.1* are located within the association signals on chromosomes 1 and 5. The black dashed lines in (c) to (f) indicate the significance threshold of GWAS associations.

**Figure 4:**
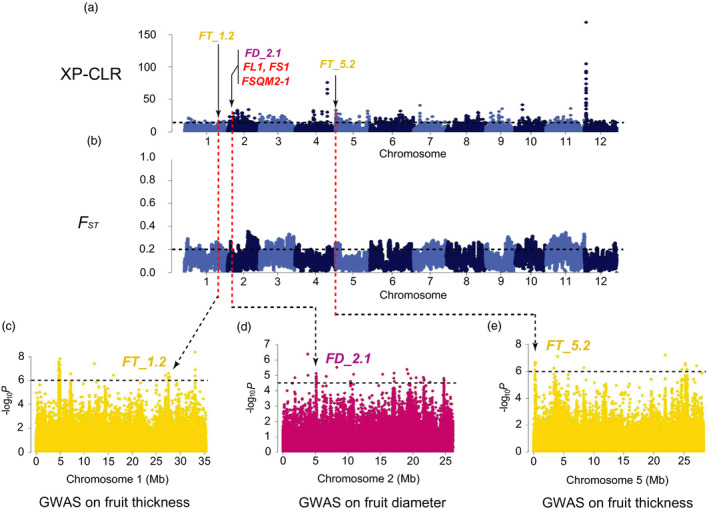
Independent selections on fruit size and flesh thickness in ssp. *agrestis* during melon breeding from landraces to improved varieties. a, Selective signals of ssp. *agrestis* in melon breeding from landraces to improved cultivars. b, Genome‐wide *F*_ST_ estimates from landraces to improved cultivars of ssp. *agrestis*. Black horizontal dashed lines indicate the genome‐wide threshold of selection signals and population differentiation index. Red horizontal dashed lines in a and b indicate the same locations between the potential selective sweeps and the population differentiation. Previously reported QTL for fruit size (red), fruit diameter (FD, dark pink) or flesh thickness (FT, yellow) and GWAS signals identified in this study overlapped with selective sweeps are marked. c to e, Manhattan plots of GWAS for flesh thickness (c, e) and fruit diameter (d). *FT_1.2*, *FD_2.1* and *FL_5.2* are located within the association signals on chromosomes 1, 2 and 5. The black dashed lines in (c) to (e) indicate the significance threshold of GWAS associations.

**Figure 5:**
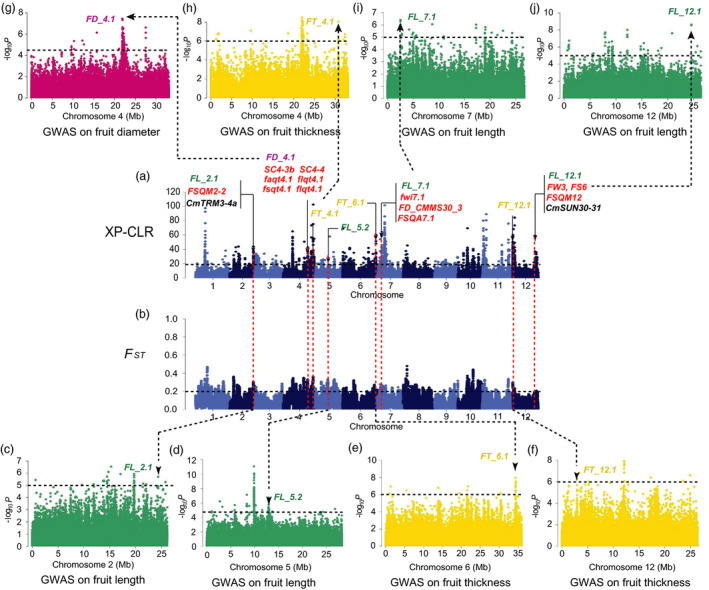
Independent selections on fruit size and flesh thickness traits from landraces to improved cultivars belonged to ssp. *melo*. a, Selective signals of ssp. *melo* in melon breeding from landraces to improved cultivars. b, Genome‐wide *F*_ST_ estimates from landraces to improved cultivars of ssp. *melo*. Black horizontal dashed lines indicate the genome‐wide threshold of selection signals and population differentiation index. Red horizontal dashed lines in a and b indicate the same locations between the potential selective sweeps and the population differentiation. Previously reported QTL for fruit size (red), fruit length (FL, green), fruit diameter (FD, dark pink) or flesh thickness (FT, yellow) and GWAS signals identified in this study overlapped with selective sweeps are marked. The fruit size homologous genes *CmTRM3‐4a* and *CmSUN30‐31* are marked with black. c to j, Manhattan plots of GWAS for fruit length (c, d, i, j), fruit diameter (g) and flesh thickness (e, f, h). *FL_2.1*, *FD_4.1*, *FT_4.1*, *FL_5.2*, *FT_6.1*, *FL_7.1*, *FL_12.1* and *FT_12.1* are located within the association signals on chromosomes 2, 4, 5, 6, 7 and 12. The black dashed lines in (c) to (j) indicate the significance threshold of GWAS associations.

Compared with wild types, landraces and improved melon cultivars have larger fruit size (FS) as reflected from the fruit length (FL) and fruit diameter (FD), which are the results of selection during domestication and breeding (Diza *et al*., 2017; Pitrat, 2013). From GWAS analysis, 489 and 393 SNPs (Tables S11 and S12, Figure S6a and b) were significantly associated with FL (‐log_10_ *P* value > 5.0) and FD (‐log_10_ *P* value > 4.5), respectively. Our recent work (Pan *et al*., 2020a) inferred 12 consensus FS and 16 fruit shape index (FSI) QTLs in melon from more than 208 published fruit size/shape QTLs (Perin *et al*., 2002; Monforte *et al*., 2004; Eduardo *et al*., 2007; Paris *et al*., 2008; Lu *et al*., 2009; Harel‐Beja *et al*., 2010; Tomason *et al*., 2013; Diaz *et al*., 2014; Ramamurthy and Waters, 2015; Perpina *et al*., 2016; Wang *et al*., 2016; Diaz *et al*., 2017; Gur *et al*., 2017; Chang *et al*., 2017; reviewed in Pan *et al*., 2020a). We compared the locations of these QTLs with our GWAS results and found 99 reported QTLs overlapped with our FL and FD GWAS signals (Table S12). We detected 246 and 108 new SNPs associated with FL and FD, respectively (Table S11). The GWAS signals for FL and FD were also estimated for XP‐CLR, *F*_ST_, π and Tajima’s *D* values aiming to detect the signals under the potential selective sweeps. We found 8 strong FS GWAS signals overlapping with selective sweeps (Figures 3, 4, 5, Figures S7‐S14), in which the locations of 6 FS GWAS signal (*FD_1.1*, *FL_2.1*, *FD_4.1*, *FL_7.1*, *FL_12.1* and *FD_2.1*) were consistent with 4 consensus FS QTLs (*CmFS2.2*, *CmFS4.2*, *CmFS7.1* and *CmFS12.2*) and 4 consensus FSI QTLs (*CmFSI1.2*, *CmFSI2.2*, *CmFSI7.1* and *CmFSI12.2*) including 18 previously reported FS QTLs (Table S13). The other 2 FS sweeps (*FL_5.1* and *FL_5.2*) were newly detected (Table S14). *FD_1.1* (in domestication stage, Figure 3a, d) overlapped with *FSQM1* (Gur *et al*., 2017) and *fs1.2* (Paris *et al*., 2008) were for ssp. *agrestis,* whereas *FD_2.1* (in improvement stage, Figure 4a and d) was consistent with previously reported *FL1*, *FS1* (Ramamurthy and Waters, 2015) and *FSQM2‐1* (Gur *et al*., 2017). Four FS selective sweeps (*FL_2.1*, *FD_4.1*, *FL_7.1* and *FL_12.1*, Figure 5a) in ssp. *melo* improvement stage shared the same regions as 13 reported QTLs: *faqt4.1*, *flqt4.1*, *fdqt4.1*, *fsqt4.1*, *fwqt4.1* (Diaz *et al*., 2017), *FSQM2‐2*, *FSQM12*, *FSQA7.1* (Gur *et al*., 2017), *FW3*, *FS6* (Ramamurthy and Waters, 2015), *fwi7.1* (Harel‐Beja *et al*., 2010), *SC* *4‐3b* and *SC4‐4* (Eduardo *et al*., 2007). Most previous studies used mainly ssp. *melo* accessions for FS QTL detection. The large amount of ssp. *agrestis* accessions employed in our study helped the identification of novel ssp. *agrestis* FS loci. *FL_5.2* (Figure 5a and d) was a novel FS selective sweep (for ssp. *melo* improvement stage) that did not overlap with any reported QTLs. *FL_5.1* had the strongest GWAS signal and was unique to ssp. *agrestis* in domestication stage (Figure 3a and f). These data suggested that, while fruit size had undergone substantial increase due to selection during domestication from wild types to landraces, this trait continued to be the main target of selection during the improved stage. The FS sweeps between the two subspecies were very different indicating independent selections in the two melon groups.

In plants, members from six gene families (*SUN*, *OFP*, *OVATE*, *CNR*, *CYP78A* and *TRM*) have been shown to play important roles in regulation of fruit size and shape (Rodriguez *et al*., 2011; Monforte *et al*., 2014; van der Knaap *et al*., 2014; Diza *et al*., 2014; Diaz *et al*., 2017; Wu *et al*., 2018; reviewed in Pan *et al*., 2020a). In the melon genome, there were 72 homologs of the six gene families (Pan *et al*., 2020a). Our fruit size GWAS signals contained 19 FS homolog genes, which were co‐localized with 26 previously reported FS QTLs (Tables S15 and S16, Figure S6a and b). Two potential FS candidate genes, *CmTRM3‐4a* (*MELO3C017231*) and *CmSUN30‐31* (*MELO3C002201*) located in the improved sweeps *FL_2.1* and *FL_12.1* (Figure 5a, Figures S7 and S8), corresponded with the consensus QTL *CmFS2.2* (Paris *et al*., 2008; Tomason *et al*., 2013; Ramamurthy and Waters, 2015; Pereira *et al*., 2018; Galpaz *et al*., 2018; reviewed in Pan *et al*., 2020a) and *CmFS12.2* (Eduardo *et al*., 2007; Diaz *et al*., 2014; Ramamurthy and Waters, 2015; reviewed in Pan *et al*., 2020a), respectively (Table S15).

Flesh thickness (FT) was another important domesticated trait during melon evolution affected fruit size (Diaz *et al*., 2017; Pitrat, 2013). Our GWAS results identified 253 SNPs significantly associated with FT (‐log_10_ *P* > 6.0; Figure S6c and Table S11). Alignment of these SNPs against the XP‐CLR, *F*_ST_ and Tajima’s *D* results revealed 2 domestication and 5 improved FT selective sweeps (Figures 3, 4, 5, Table S14 and Figures S15‐S21) which were quite different between the two subspecies. So far, 5 QTLs for flesh thickness and pulp area have been reported (Diaz *et al*., 2017; Obando *et al*., 2008) which were located on chromosomes 5 (*paqt5.1*, *ptqt5.1*), 6 (*paqt6.1*) and 8 (*paqt8.1*, *ptqt8.1*). Among them, *paqt6.1* overlapped with our results (Figure S6c). The FT GWAS signal on chromosome 5 was located in a region that is syntenic to cucumber chromosome segment harbouring *Csa2M058670.1* that is the candidate gene for cucumber flesh thickness (Xu *et al*., 2015a). The homolog of cucumber *Csa2M058670.1* in melon, *MELO3C004029*, is also located in this region. Expression of this gene peaks near the mature stage during fruit development in melon flesh and rind, and exhibits decreased expression when the fruit is fully ripen (from published RNA‐seq data; Figure S22). In most melon accessions, the FT of ssp. *agrestis* was significantly thinner than that of ssp. *melo*. Three selective sweeps for FT from the LDR_M to IMP_M were detected, only 2 of which were found from LDR_A to IMP_A, suggesting more intense selection for thicker flesh in ssp. *melo* in the improved stage. FS and FT were selected in both the domesticated and improved stages, suggesting a two‐step selection with different loci in the two subspecies.

### GWAS for carpel number (CN) and its pleiotropic effects for fruit shape

**Figure 6:**
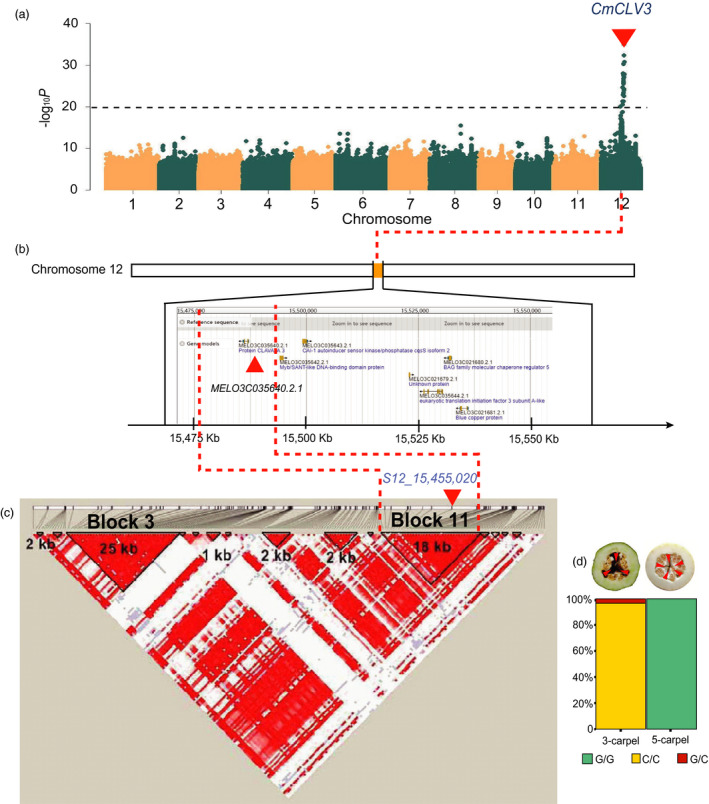
*Clavata3* (*CmCLV3*) is the candidate gene underlying carpel number variation in melon. a, Manhattan plots of GWAS for carpel numbers in melon accessions. The black dashed lines indicate the significance threshold of association. b, The location of GWAS‐associated region on chromosome 12 and candidate genes. The red triangle indicates *CmCLV3* (*MELO3C035640.2*). c, LD block analysis for the GWAS region and the 18 kb (Block 11) segment harbouring *CmCLV3* and co‐segregating SNP (*S12_15,455,020*). The red triangles in (a), (b) and (c) indicate the locations of *CmCLV3*, *MELO3C035640.2* and the co‐segregating SNP (*S12_15,455,020*), respectively. d, The genotypic frequencies at SNP locus *S12_15,455,020* in 3‐carpel and 5‐carpel melon accessions. Fruits of representative 3‐carpel and 5‐carpel melon accessions are shown on the top of (d). The red triangles indicated the locations and numbers of carpels.

Many factors regulate FS in plants including carpel number, which has been shown to be controlled by *CLAVATA3* (*CLV3*) in tomato and cucumber (Li *et al*., 2016; Pan *et al*., 2020a; Pan *et al*., 2020b; Xu *et al*., 2015b). Among 245 accessions in our study, 204 and 41 had 3 and 5 CN, respectively. GWAS in this population revealed a strong signal for CN on chromosome 12 (Figure 6a), which spanned 113.91 kb (chromosome 12: 15 398 970 to 15 512 877) with 5 predicted genes in the DHL92 V3.5.1 genome (*MELO3C021677*, *MELO3C021678*, *MELO3C021679*, *MELO3C021680* and *MELO3C021681*). LD analysis with 149 accessions and 292 SNPs revealed 15 LD blocks in this GWAS region. *MELO3C021677* (annotated as UTP‐glucose‐1‐phosphate uridylyltransferase) was the only gene in Block 3 but without any sequence or gene structure variation associated with carpel number among the melon accessions. The other 4 genes were not located in any block. After comparing the genotypic data and carpel number, we found one SNP (*S12_15,455,020*, *P* value = 3.955E‐36) in Block 11 that was perfectly co‐segregating with carpel number (Figure 6b and c, Table S12). In the most recent DHL92 V3.6.1 (chromosome 12: 15 430 184 to 15 534 928), the 113.91kb contained 7 annotated genes including 3 in DHL92 V3.5.1 (*MELO3C021679*, *MELO3C021680* and *MELO3C021681*) and 4 were newly annotated (*MELO3C035640.2.1*, *MELO3C035643.2.1*, *MELO3C035642.2.1* and *MELO3C035644.2.1*, Figure 6b). *MELO3C035640.2.1* was predicted to encode *CmCLV3* that harbours *S12_15,455,020* in Block 11. Two alleles could be detected at *S12_15,455,020* locus among our GWAS panel: *S12_15,455,020^C/C^* and *S12_15,455,020^G/G^* that corresponds to 3‐carpel and 5‐carpel lines, respectively (Figure 6d). *S12_15,455,020^C/G^* have fruits with 3 carpels, which is consistent with the dominant nature of 3‐carpel to 5‐carpel. In cucumber, *CsCLV3* is a major regulator of carpel numbers and 3‐carpel is dominant to 5‐carpel (Li *et al*., 2016). Thus, our results revealed the structure and function conservation of the *CLV3* gene in carpel number determination in the two species (Pan *et al*., 2020b). The region harbouring *CmCLV3* is syntenic of cucumber chromosome 1 (Gy14v2.0) where *CsCLV3* was located (Li *et al*., 2011; Pan *et al*., 2020b; Yang *et al*., 2014). Comparative gene expression analysis between 3 and 5 carpel number melon accessions showed that *CmCLV3* had a high expression in the early stage of ovary development and then declined. Meanwhile, its expression in 3‐carpel melons was significantly higher than in 5‐carpel ones at the ovary stage (Figure S23a). These data strongly support *CmCLV3* as the candidate gene for melon carpel number variation.

Previous studies identified melon FS QTLs such as *fdqs12.1*/*fsqs12.2* (Diaz *et al*., 2014), *FSQM12* (Gur *et al*., 2017) and some of our GWAS signals which were also co‐localized with the *CmCLV3* region (Table S12) suggesting that carpel number may also affect fruit shape as found in cucumber (Li *et al*., 2016; Pan *et al*., 2020b). We further analysed the relationship of carpel numbers, and FSI in 149 accessions from our GWAS panel (Table S17). Two 3‐carpel accessions that belonged to the *C. melo* ssp*. flexuosus* group (x091 and m4‐75) with very long fruit (67.5 ± 1.43 cm and 44.5 ± 2.51 cm, respectively) were excluded in this comparison to avoid excessive effect of the extreme FL. In 2015 and 2016 field trials, the mean FSI of 117 3‐carpel accessions was 1.43 ± 0.42 and 1.46 ± 0.50, while those for the 32 5‐carpel were 1.07 ± 0.18 and 1.06 ± 0.18, respectively, indicating 5‐carpel fruits tend to be rounder than 3‐carpel fruits. In melon and cucumber, plants with andromonoecious or hermaphroditic sex expression often produce round fruits (Boualem *et al*., 2008; Pan *et al*., 2020b; Tan *et al*., 2015; Xin *et al*., 2019). This interesting relationship may be due to the pleiotropic effects of the *andromonoecy* (*a*/*CmACS7*) gene in melon and the *m*/*CsACS2* gene in cucumber, which is probably related to the ethylene accumulation level, rather than the tight linkage to FS genes (Boualem *et al*., 2008; Galpaz *et al*., 2018). However, in melon, the relationship between *CmACS7* and *CmCLV3* for FS is not clear. Hence, 123 out of 149 accessions were phenotyped for sex expression to determine possible effects of *CmCLV3* on FS. Of the 123 accessions, 104, 18 and 1 were andromonoecious, monoecious and trimonoecious, respectively. Among 28 5‐carpel accessions, 27 were andromonoecious. The FSI of 5‐carpel andromonoecious lines in 2015 and 2016 was 1.06 ± 0.17 and 1.06 ± 0.18, respectively, suggesting that all have round fruits. Among the 95 3‐carpel accessions, 77, 17 and 1 were andromonoecious, monoecious and trimonoecious, respectively. The FSI of the 77 3‐carpel andromonoecious accessions was 1.78 ± 0.69 and 1.70 ± 0.60 in 2015 and 2016, respectively, while FSI for the 17 3‐carpel monoecious melons was 1.36 ± 0.37 and 1.35 ± 0.36 (Figure S23b to d). As compared with 3‐carpel monoecious melons, 3‐carpel andromonoecious ones had longer FL, but for the FD, the variation among different genotypes with 3‐ and 5‐carpel melon accessions was not significant. These results suggested that *CmCLV3* might still affect FSI in the andromonoecious genetic background. Of 117 3‐carpel lines, 35 had FSI similar to that of 5‐carpel accessions; conversely, 8 out of 32 5‐carpel accessions had FSI values close to the 3‐carpel lines, which may be caused by other FS loci.

## Discussion

### Independent and two‐step selections in the ssp. *melo* and ssp. *agrestis* lineages of melon

Wild melons, sometimes called ‘weed melon’ (Pitrat, 2013), were found in Africa and Asia, while the sister species of *C*. *melo* were found in Australia (Sebastian *et al*., 2010; Telford *et al*., 2011). Both Africa and Asia were proposed as the centres of diversity for melon (Kerje and Grum, 2000; Kirkbride, 1993; van Zeist and Roller, 1993; Zhao *et al*., 2019). Wild melon and landraces from India and East Asia exhibited high genetic diversity (Dhillon *et al*., 2007; Dwivedi *et al*., 2010; Roy *et al*., 2011), which was confirmed by our population genetic analysis. Zhao *et al*. (2019) suggested two independent domestication events for ssp. *melo* and ssp. *agrestis* that occurred in India. Our data presented herein support this notion. Based on the results of ML phylogenetic tree, PCA, LD decay and population structure analyses (Figures 1 and 2), the landraces were clearly separated from the improved varieties. The π value was 0.98 × 10^−3^ in LDR_A that was sharply decreased to 0.28 × 10^−3^ in IMP_A suggesting significant reduction in the genetic diversity during the improvement from LDR_A to IMP_A. The rates of LD decay between landrace and improved cultivars were also significantly different in the two subspecies. These implied that the improved stage played an important role in melon breeding, especially for ssp. *agrestis*. Based on these, we further found the different diversification selection histories in the two subspecies from landraces to improved cultivars. Different signals between domesticated and improved stages for each subspecies were also detected. The selective signals were different between WT versus landrace as well as landrace versus improved cultivar (Figures 3, 4, 5) indicating the important role of landraces in melon crop evolution. We calculated *F*_ST_ and XP‐CLR of ssp. *agrestis* WT versus LDR_M and detected signals between the two groups. More than half of the total XP‐CLR regions between WT and LDR_A could be detected in WT versus LDR_M. This may imply that in the domesticated stage, a lot of chromosomal regions were under selection in both subspecies. The strong differentiation may occur during the improved stage. Most previous genetic diversity studies used materials from ssp. *melo* with very few of ssp.*agrestis*, especially for *chinensis* and *makuwa*. Most horticulture groups in ssp.*agrestis* are cultivated in China. Our GWAS panel included 58.9% ssp. *agrestis* and 41.1% ssp. *melo* accessions. In this sense, our work has provided unique perspectives on the evolution of ssp. *agrestis*, which were not available in previous studies.

Interestingly, two ‘Mapao’ melon accessions (*Cucumis melo* L. var. *agrestis* Naud.) (‘x207’ and ‘1114wd’, Table S1) have long been thought to be wild melon in China (Lin, 1984), but they were associated with the LDR_A and IMP_A in the ML tree and PCA plot. Population structure analysis also revealed only trace of the WT ancestries in both accessions (Figure S1) implying a much more domesticated status of the Mapao melon. This is interesting since the plants and mature fruits of the ‘Mapao’ melons used in the present study were morphologically similar to other wild melons, such as small leaves, flowers, stem, fruits and small seeds with a gelatinous sheath except for the yellow skin and andromonoecious sex expression. China was regarded as the secondary diversity centre owing to the high genetic diversity of *C*. *melo* landraces (Luan *et al*., 2008; Sebastian *et al*., 2010). The ‘Mapao’ melons are morphologically more similar to those in *chito* melon group cultivated in Central America and the Caribbean Islands that are considered as feral melon (Pitrat, 2013 and Pitrat, 2016). Whether the Mapao melon is a feral form of cultivated melon required further investigation.

### Two *AAT* genes may contribute to the unique aroma in melon

Aroma is an important trait that has been under selection in melon breeding (Pitrat, 2013). *Cm* *AATs* play an important role in the last step of ester biosynthesis, which results in synthesis of variety esters for aroma in melon (Chen *et al*., 2016; Galaz *et al*., 2013; Oh *et al*., 2011). In this study, a chromosomal region containing five *CmAATs* was detected with a high selective signal. Expression of three of the five *CmAAT* genes (*MELO3C024766*, *MELO3C024769* and *MELO3C024771*) was highly correlated with aroma level in mature fruit. High level of alcohols and the volatile esters in melon is correlated with *CmAAT1* (*MELO3C024771*) activity (El‐Sharkawy *et al*., 2005; Galaz *et al*., 2013). Galpaz *et al*. (2018) identified a major‐effect QTL (LOD = 4.02–5.84, *R* ^2^ = 19%–26%) for high level of alcohols and volatile esters in melon volatiles which is overlapped with *CmAAT1* (*MELO3C024771*) and *CmAAT2* (*MELO3C024766*). The total enzymatic activity of CmAAT (CmAAT1 and CmAAT2) increases as melon fruit ripens, which is positively correlated with the ester content in strong‐aromatic and less‐aromatic melons, but no such change in its activity in non‐aromatic ones that always have very low enzyme activity (Chen *et al*., 2016). *CmAAT2* was strongly expressed near melon maturation and also for the enzyme active (Guo *et al*., 2019). The diversity of volatile esters generated from multiple AAT enzymes with a different specific substrate selectivity (D’ Aurian *et al*., 2006; Lucchetta *et al*., 2007). Synthesis of major volatile esters in melon, such as E‐2‐hexenyl acetate, hexyl hexanoate, benzyl acetate and cinnamyl acetate, is catalysed by different CmAATs (El‐Sharkawy *et al*., 2005). Data from previous studies do support *CmAATs* as strong candidates for aroma accumulation in melon, which is consistent with our findings in this study. In comparison with the early work (Zhao *et al*., 2019), *MELO3C024762*, *MELO3C024764* and *MELO3C024771* were present in the selective regions in both ssp. *melo* and ssp. *agrestis* suggesting aroma accumulation may have undergone parallel selections in the two subspecies. Based on the expression pattern of public RNA‐seq data, *MELO3C024771* (*CmAAT1*) exhibited the highest expression during melon fruit development in multiple accessions than all other *CmAATs* suggesting its important role in aroma generation. The CmAAT1 protein consists of two approximately equal‐sized domains connected through a large crossover loop (Galaz *et al*., 2013). The highly correlated SNP (*S11_8043933*) was located in the genic DNA region for the connecting loop of CmAAT1 protein structure. These indicated that *S11_8043933* may be an important mutation under selection during melon domestication. Previous studies found that mutation in *CmAAT2* caused by amino acid substitution at 268th position (T268 → A268) will result in no production of volatile esters (El‐Sharkawy *et al*., 2005; Galaz *et al*., 2013). In our GWAS panel, we did not find association of this SNP with aroma production. Thus, although this locus plays an important role for the CmAAT protein activity, the amino acid substitution may not happen during melon evolution. The higher correlation of *S11_7918549* (I372 → D372) implied an important role of *CmAAT2* function during melon domestication, but as previously reported, this SNP is only located in the coding region but not located in any functional domain in CmAAT2 protein structure (Galaz *et al*., 2013). Nevertheless, *S11_7918549* may provide new information of the *CmAAT2* gene function. The SNP variation of *CmAAT1* and *CmAAT2* (Table S10) could also be used in molecular breeding. Compared with other cucurbits, melon produces unique aroma in mature fruit, which might be attributed to the unique presence of *MELO3C024771* and *MELO3C024769* in the melon genome. But further work is needed to support this.

### Selective sweeps for fruit size during two‐step selections

We identified 15 selective sweeps associated with FS and FT. Seven of them (*FD_1.1*, *FD_2.1*, *FL_5.1*, *FT_1.1*, *FT_1.2*, *FT_5.1* and *FT_5.2*) were also detected as putative domestication sweeps by Zhao *et al*. (2019) but with no association of particular traits. We found that these FS and FT sweeps were different in the two stages for two subspecies supporting two‐step and independent selections in each lineage. This finding was supported by FS QTL detected in previous QTL mapping studies using bi‐parental populations (Table S12). For example, the four improved sweeps, *FL_2.1*, *FD_4.1*, *FL_7.1* and *FL_12.1* in ssp. *Melo,* were detected with segregating populations derived from crosses between a landrace and an improved variety (PI 435288 × C940‐fe, Ramamurthy and Waters, 2015; Piel de Sapo × PI 161375, Eduardo *et al*., 2007; K7‐1 × K7‐2, Lu *et al*., 2009), or between two landraces (Piel de Sapo × PI 124112, Diaz *et al*., 2014), or improved varieties (PI 414723 × Dulce, Harel‐Beja *et al*., 2010), or a landrace and a wild melon from India (Piel de Sapo × *Trigonus*, Diaz *et al*., 2017). For the four FS sweeps, each sweep contained at least one overlapped QTL detected in the genetic backgrounds between a landrace and an improved variety, thus proving evidence for the landrace‐improved hypothesis. Interestingly, all the parental materials in the above‐mentioned QTL mapping populations belong to ssp. *melo* corresponding to the comparison between LDR_M and IMP_M in our study. For ssp. *agrestis*, we found one improvement sweep (*FD_2.1*) and one domestication sweep (*FD_1.1*) that were overlapped with published QTLs. *FD_2.1* seems to correspond to *FSQM2* that was detected from a GWAS panel with 177 melon accessions including 28 ssp. *agresits* (Gur *et al*., 2017). *FD_1.1* was overlapped with both *FSQM1* (Gur *et al*., 2017) and *fs1.2* (Paris *et al*., 2008). The parental line USDA 864‐1 carrying *fs1.2* was derived from CR‐1 (ssp. *agrestis*). The ancestry composition of the experimental materials in the two populations may suggest that the two sweeps are unique to the ssp. *agrestis* lineage.

## Methods

### Germplasm resources and phenotypic data collection

Two hundred and ninety‐seven melon accessions were used for resequencing and phenotypic data collection (Table S1). These melon lines belong to two subspecies, ssp.*agrestis* and ssp.*melo*, which could be further classified into 5 subgroups including 16 WT (wild types), 50 LDR_A (landrace ssp. *agrestis*), 41 LDR_M (landrace ssp. *melo*), 109 IMP_A (improved ssp. *agrestis*) and 54 IMP_M (improved ssp. *melo*) according to the plant introduction information. The improved status of 27 accessions was unknown (ND, not defined). The accessions were evaluated in a randomized complete block design with three replications during a 2‐year period (2015 and 2016) in the Xiangyang Experimental Farm, Northeast Agricultural University, Harbin (46° 40′ N125° 42′ E), China. Each accession had 5 plants with 50 × 30 cm spacing. Each plant was self‐pollinated to generate 2–4 fruits. Phenotypic data including fruit length, diameter, flesh thickness and carpel number were recorded for each fruit. Fruit size and flesh thickness were evaluated with ruler on the longitudinal direction on mature fruits. At least three ovaries were surveyed for carpel number on each plant.

### DNA extraction and whole‐genome resequencing

Equal amounts of young true leaves were collected from five individuals of each accession was collected and mixed equally for genomic DNA extraction using the CTAB method. Paired‐end sequencing libraries (150 bp × 2) were constructed for all the 297 lines following manufacturer’s instructions (Illumina). The libraries were sequenced in Illumina Genome Analyzer X10 platform by BGI, China. The melon reference genome DHL92 V3.5.1 (Garcia‐Mas *et al*., 2012) and its annotation were downloaded online (http://cucurbitgenomics.org). All resequencing data were deposited in GenBank (https://www.ncbi.nlm.nih.gov/) under BioProject ID PRJNA529037.

### SNP calling

Three steps were taken to generate the SNP‐genotype variant call format (VCF) file for the 297 melon accessions. The clean reads were mapped to the melon reference genome using BWA v0.7.12 (Li and Durbin, 2009) with following parameters: ‘‐m 200000 ‐o 1 ‐e 30 ‐i 15 ‐L ‐I ‐t 4 ‐n 0.04 ‐R 20’ to get read‐mapping SAM files. SAMtools (Li *et al*., 2009) was used to convert SAM files into BAM and the indexed BAM files. The Genome Analysis Toolkit (GATK, V 3.3‐0; McKenna *et al*., 2010) was then used to call high‐quality SNPs with the following criteria: (i) duplicate reads from PCR were removed by Picard package V1.105; (ii) base quality score were recalibrated by Base Recalibrator package in GATK; (iii) SNPs were called using the Unified Genotyper function in GATK with parameters as follows: 50 of minimum phred‐scaled confidence value and 10 of minimum phred‐scaled confidence threshold; (iv) low‐quality SNPs were filtered using the variant filtration function in GATK with default parameters; and (v) variant quality was assessed using the Variant Recalibrator and Apply Recalibration functions with 99 of truth sensitivity filter level in GATK with the parameters.

### Phylogenetic and population structure analysis

The 18 510 SNPs were selected from the entire fourfold degenerate codon transversion (4DTv) SNP data to develop the maximum‐likelihood (ML) tree using IQ‐TREE (Nguyen *et al*., 2015) with 1000 bootstraps (parameters: ‐nt 5 ‐m GTR ‐bb 1000). The SNP‐genotype VCF file of 297 melon accessions was converted into a matrix by PLINK 1.90 (Purcell *et al*., 2007) with the function of ‐‐make‐bed (parameters: ‐‐geno 0.05 and ‐‐hwe 0.0001) for PCA. The eigenvector decomposition of the matrix was estimated by GCTA 1.26.0 (Yang *et al*., 2007) with the function of ‐‐make‐grm and ‐‐autosome. Population structure was analysed using ADMIXTURE 1.3.0 (Alexander *et al*., 2009). The initialization of population number (*K* value) was set from 2 to 7 to determine the minimum estimated crossed‐validation error (parameters: ‐‐geno 0.05 ‐‐maf 0.05 ‐‐hwe 0.0001). The ML tree was coloured with iTOL (https://itol.embl.de). The PCA plots and population structure graph were drawn with R packages and Origin 2018, respectively. Linkage disequilibrium (LD) was estimated and plotted using SNP data with MAF (minor allele frequency) > 0.05 by PopLDdecay3.31 (Zhang *et al*., 2018) for each subpopulation across the whole genome. The physical distance of LD decay for each subgroup was estimated with a cut‐off value at *r* ^2^ = 0.25.

### Identification of selective sweeps

A whole‐genome scan was performed using an updated cross‐population composite likelihood approach with XP‐CLR 1.0 software (Chen *et al*., 2010). Selection sweeps across the genome were evaluated in the following pairs of data sets: WT versus LDR_A; WT versus LDR_M; LDR_M versus IMP_M; and LDR_A versus IMP_A. A 0.05‐cM sliding window with 100‐bp steps across the whole genome was used for scanning. To ensure comparability of the composite likelihood score in each window, the maximum number of SNPs in each window was set to 200. Individual SNPs were assigned to positions along an ultra‐dense genetic map (Hu *et al*., 2018) by assuming uniform recombination between mapped markers. The command line was XPCLR ‐xpclr InputFile1 InputFile2 mapFlie outputFile ‐w1 0.005 100 200 chrN ‐p0 0.95. The likelihood score in 50‐kb sliding windows was averaged with a step size of 5 kb across the genome. Adjacent windows (<50 kb) with high XP‐CLR were merged into a single region to represent the effect of a single selective sweep and exhibited in the forms of Manhattan plots with R packages. The top 5% highest XP‐CLR values were considered as selected regions.

### Genetic diversity and population differentiation

Genotypes and SNP positions were used to estimate the nucleotide diversity (π) with a step size of 5 kb and in 50‐kb sliding windows and Tajima’s *D* with the function of ‐‐window‐pi and –Tajima’s D in each subpopulation using VCFtools 0.1.16 (Danecek *et al*., 2018). The plots of π and Tajima’s *D* were drawn using OmicShare tools (http://www.omicshare.com/tools/). The coefficient of divergence index (*F*_ST_) value between two populations was estimated with 50‐kb sliding windows and a 5‐kb step size across the genome using the function of weir‐fst‐pop in VCFtools 0.1.16 to identify the chromosome regions with high differentiation between different subgroups. Windows with the top 5% of mean *F*_ST_ values were selected and merged into regions as the key divergent regions presented with Manhattan plots with R packages.

### Genome‐wide association mapping

GWAS analysis for fruit length, fruit diameter, flesh thickness and carpel number was performed with 2 045 412 high‐quality SNPs (MAF > 0.05). The compressed general linear model (GLM) and compressed mixed linear model (MLM) were used. *P*‐values of association of each SNP with individual trait were calculated with TASSEL 5.0 (https://tassel.bitbucket.io/).

### Published RNA‐seq data and *CmCLV3* expression analysis

The publicly available genome assemblies and RNA‐seq data (http://cucurbitgenomics.org/) from different cucurbit crops were used for comparative analysis and evaluation of gene expression patterns in target regions. The BioProject numbers of RNA‐seq data sets were PRJNA286120, PRJNA288543, PRJNA314069 and PRJNA383830 for melon; PRJNA221197, PRJNA270773, PRJNA338036 and SRP012849 for watermelon; PRJNA312872 for cucumber; PRJNA385310 for *Cucurbita maxima* (*Rimu*) and *Cucurbita moschata* (*Rifu*); PRJNA339848 for *Cucurbita pepo* (*Zucchini*); and PRJNA387615 for bottle gourd. The RPKM mean value, standard deviation for each candidate gene in melon and the homologous genes in other cucurbits were retrieved from above‐mentioned data sets. Significance of differential gene expression in different samples was tested with one‐way ANOVA in SPSS 23 software. The results were plotted with the software of GraphPad Prism 8.

For quantitative real‐time PCR of *CmCLV3*, three ovaries from two melon accessions: M1‐7 (5 carpels) and M1‐96 (3 carpels) were collected at three development stages (2 mm, 4–5 mm ovary length and the ovary at anthesis) and flash‐frozen in liquid nitrogen. Total RNA was extracted using RNA extraction kit (Novogene, Beijing). The cDNA was synthesized using the ReverTra Ace qPCR RT Kit (Toyobo, Japan). The primers used for *CmCLV3* expression analysis were 5′‐AGATAAGGGCGGGAAGAGGT‐3′ and 5′‐TGATGCAATGGGTCAGGTCC‐3′. The expression of *MELO3C023264* (actin, 5′‐TGCCCAGAAGTTCTATTCCAGC‐3′ and 5′‐CATAGTTGAACCACCACTGAGGAC‐3′) was used as the internal control. The reaction was performed using the QTOWER Real‐Time PCR System (Analytik Jena, Germany) with SYBR Green Master Mix (Novogene, Beijing). Negative controls with no cDNA templates were included in all runs to screen for potential contamination. Samples of M1‐96 at 2 mm ovary length were used for calibration. The relative expression was analysed using the 2^−ΔΔCT^ method.

## Conflict of interest

The authors declare that they have no competing interests.

## Authors’ contributions

F.S. and Y.W supervised the project and participated in writing and revision of the manuscript. S.L. and P.G. designed the experiments and performed the studies. S.L. prepared the draft manuscript. Q.Z. participated in bioinformatic analysis. Z.Z., H.L., X.W. and M.G. participated in sample preparation and phenotypic data collection. S. L. and P. G. contributed equally to this work. All authors have read and approved the final manuscript.

## Supporting information


**Figure S1** Population structure among the 14 wild types and two “Mapao” melon accessions.
**Figure S2** Independent selection compared with ssp. *agrestis* wild melon to ssp. *melo* landrace.
**Figure S3** The putative aroma selective sweep conferred *CmAAT*s.
**Figure S4** Expression pattern analysis of *CmAAT*s with published RNA‐seq data.
**Figure S5** Expression pattern analysis of *CmAAT*s with published RNA‐seq data in cucumber and *Cucurbita moschata* (*Rifu*).
**Figure S6** Manhattan plots of GWAS for melon fruit length, fruit diameter and flesh thickness.
**Figure S7** **to Figure S14** Potential fruit size selective sweeps.
**Figure S15** **to Figure S21** Potential flesh thickness selective sweeps.
**Figure S22** Expression pattern analysis of *MELO3C004029* with published RNA‐seq data during melon fruit development.
**Figure S23** *CmCLV3* expression pattern in different carpel number melon accessions during melon ovary development and the variations of fruit length, fruit diameter, fruit shape index and sex expression among different carpel number melon accessions.

**Table S1** Information of 297 melon accessions used in this study.
**Table S2** Putative regions of domestication sweeps (top 5%).
**Table S3** Putative regions of improvement sweeps (top 5%).
**Table S4** Genes within the putative improvement sweeps in ssp. *agrestis* (top 5%).
**Table S5** Genes within the putative improvement sweeps in ssp. *melo* (top 5%).
**Table S6** Genes within the putative improvement sweeps both in ssp. *agrestis* and *melo* (top 5%)
**Table S7** Putative regions of domestication with *F*_ST_ values (top 5%).
**Table S8** Putative regions of improvement with *F*_ST_ values (top 5%)
**Table S9** Overlapping regions between XP‐CLR and *F*_ST_ values.
**Table S10** Haplotype analysis of *CmAAT1* and *CmAAT2*.
**Table S11** Novel GWAS signals for fruit size and flesh thickness detected in this study.
**Table S12** Fruit size GWAS signals overlapped with the reported QTLs.
**Table S13** Fruit size sweeps overlapped with the reported QTL and consensus QTL.
**Table S14** Overlapping regions between newly detected fruit length and flesh thickness GWAS signals and putative selection sweeps.
**Table S15** Fruit size homologous genes located in the GWAS signal region overlapped with the reported QTLs.
**Table S16** Fruit size homologous genes located in the GWAS signal region (new detected).
**Table S17** Carpel number, fruit shape and haplotype of the 149 melon accessions.
