# Supplementary material for: Resequencing of 297 melon accessions reveals the genomic history of improvement and loci related to fruit traits in melon
Source: Plant Biotechnol J. 2020 Jun 30;18(12):2545–58. doi: 10.1111/pbi.13434 (PMC7680547; doi:10.1111/pbi.13434)
Supplement: Supplementary file 1 — Figure S1 Population structure among the 14 wild types and two “Mapao” melon accessions. Figure S2 Independent selection compared with ssp. agrestis wild melon to ssp. melo landrace. Figure S3 The putative aroma selective sweep conferred CmAATs. Figure S4 Expression pattern analysis of CmAATs with published RNA‐seq data. Figure S5 Expression pattern analysis of CmAATs with published RNA‐seq data in cucumber and Cucurbita moschata (Rifu). Figure S6 Manhattan plots of GWAS for melon fruit length, fruit diameter and flesh thickness. Figure S7 to Figure S14 Potential fruit size selective sweeps. Figure S15 to Figure S21 Potential flesh thickness selective sweeps. Figure S22 Expression pattern analysis of MELO3C004029 with published RNA‐seq data during melon fruit development. Figure S23 CmCLV3 expression pattern in different carpel number melon accessions during melon ovary development and the variations of fruit length, fruit diameter, fruit shape index and sex expression among different carpel number melon accessions. [file PBI-18-2545-s001.pdf]

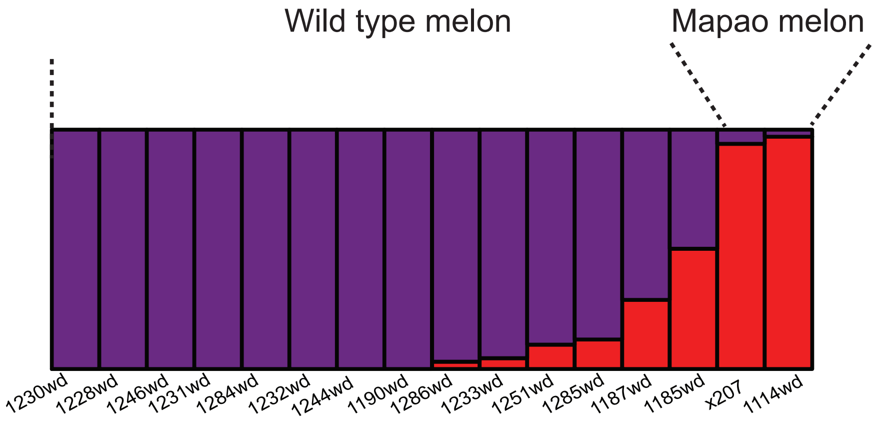

Fig. S1 Population structure among the 14 wild types and two “Mapao” melon accessions.

K = 3, the x axis lists the different accessions. Black horizontal dashed lines indicate the wild types and the “Mapao” melon.

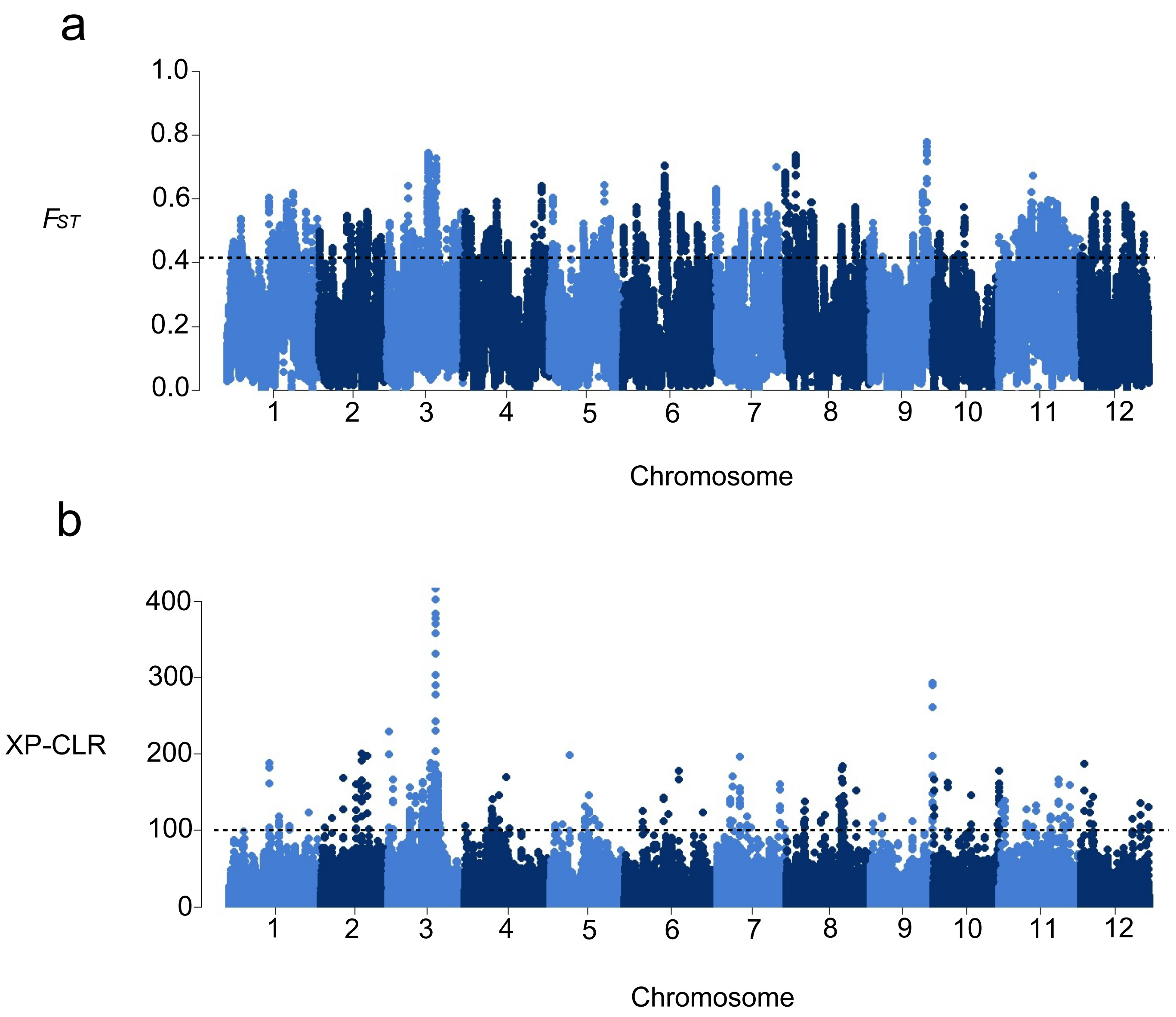

Fig. S2 Independent selection compared with ssp. agrestis wild melon to ssp. *melo* landrace.

a, Selective signals between ssp. *agrestis* wild types and ssp. *melo* landraces. **b**, F_{ST} values among the whole genome between ssp. *agrestis* wild types and ssp. *melo* landraces. Black horizontal dashed lines indicate the genome-wide threshold of selection signals and population differentiation index.

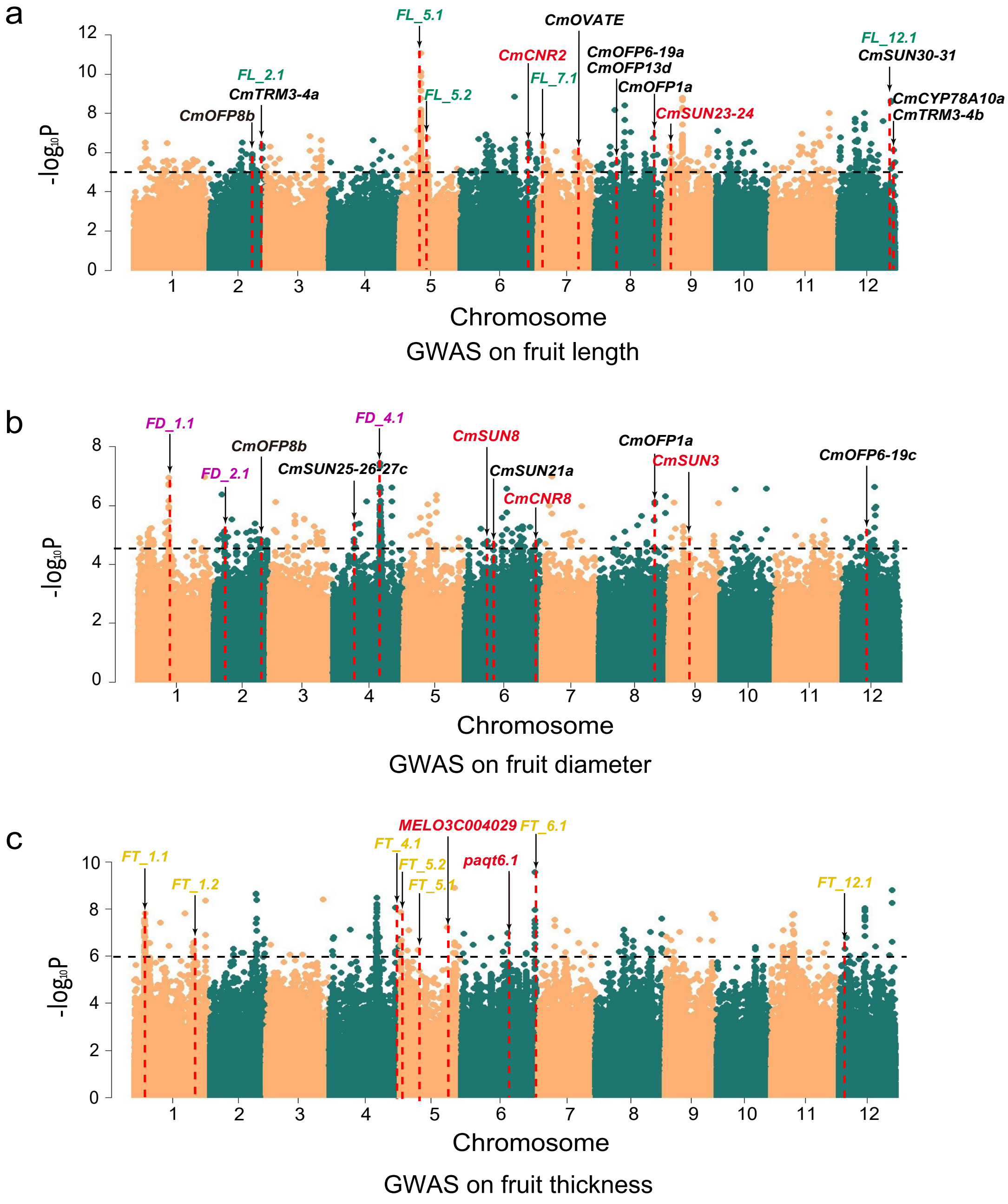

Fig. S3 Manhattan plots of GWAS for melon fruit length, fruit diameter and flesh thickness.

a, Manhattan plots of GWAS for fruit length. **b**, Manhattan plots of GWAS for fruit diameter. **c**, Manhattan plots of GWAS for flesh thickness. Black horizontal dashed lines indicate the genome-wide threshold of selection signals and population differentiation index. Red horizontal dashed lines in **a**, **b** and **c** indicate the locations of GWAS signals. The fruit size homologous genes shared the same location both with the previously reported fruit size QTLs and our GWAS results are marked with black. The fruit size homologous genes only located in our fruit size GWAS signals are marked with red. Fruit length (FL, green), fruit diameter (FD, dark pink), flesh thickness (FT, yellow) GWAS signals identified in this study are marked with different colors. *MELO3C004029* and *paqt6.1* are marked with red.

Fig. S4

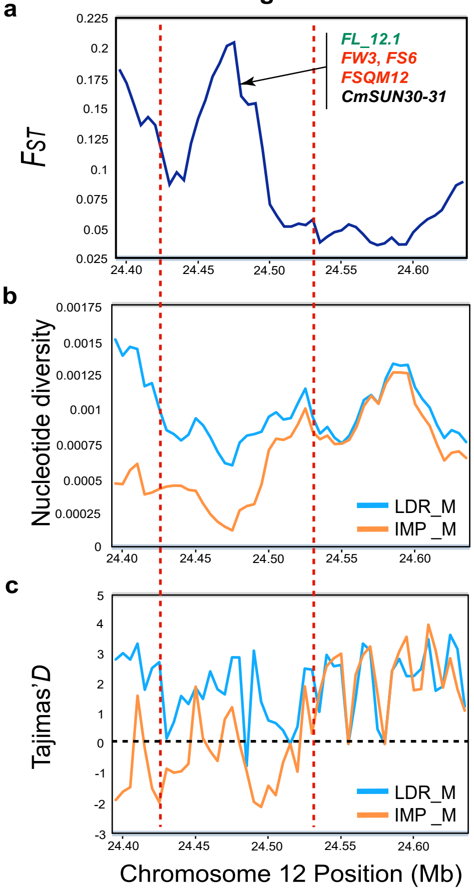

Fig. S5

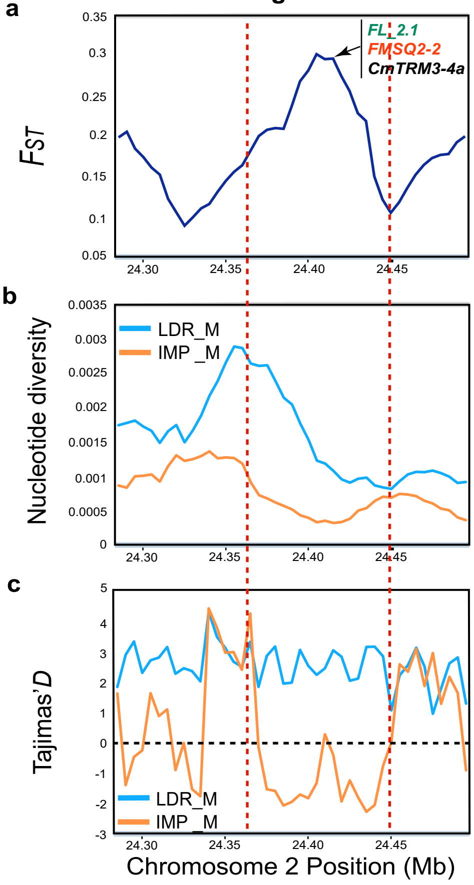

Fig. S6

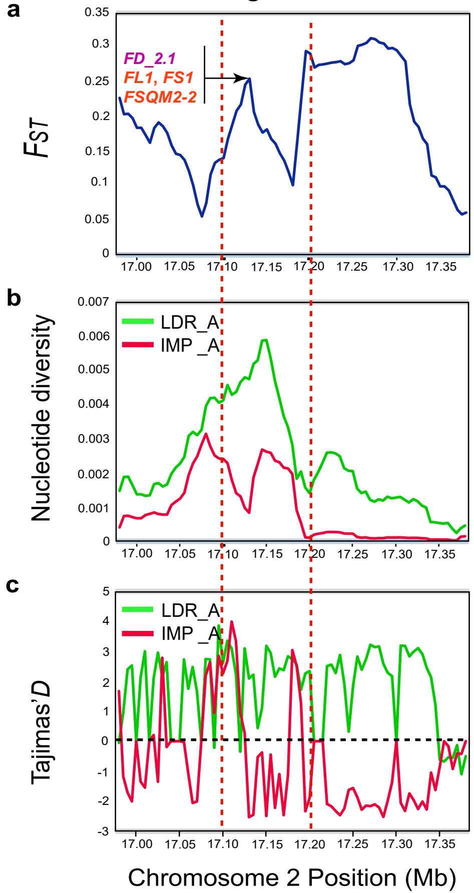

Fig. S7

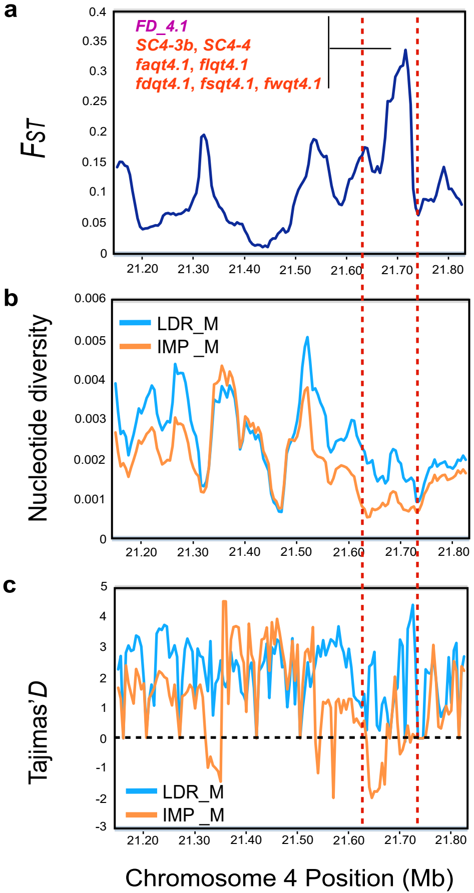

Fig.S8

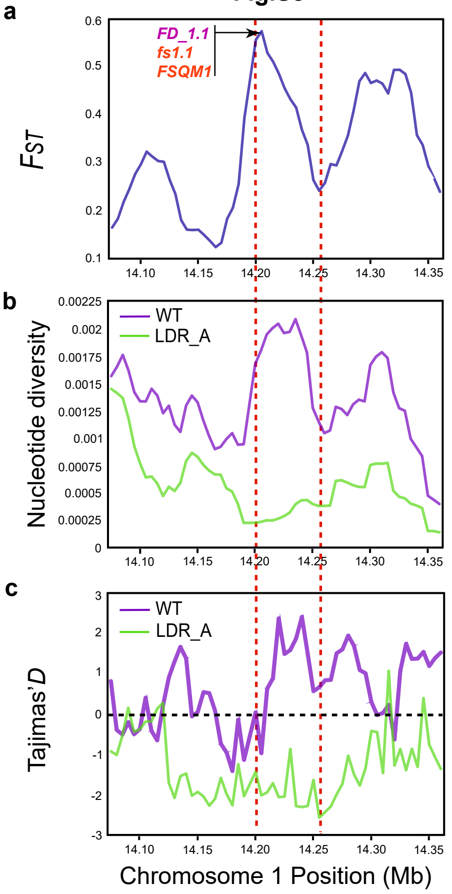

Fig. S9

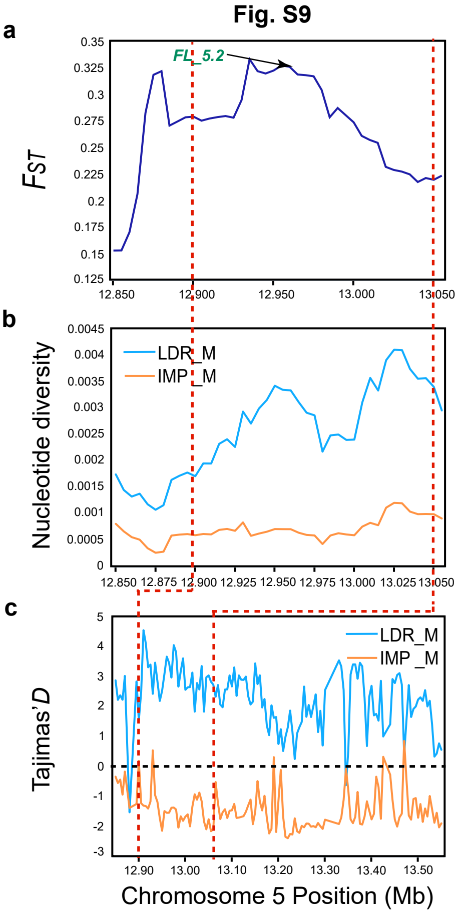

Fig. S10

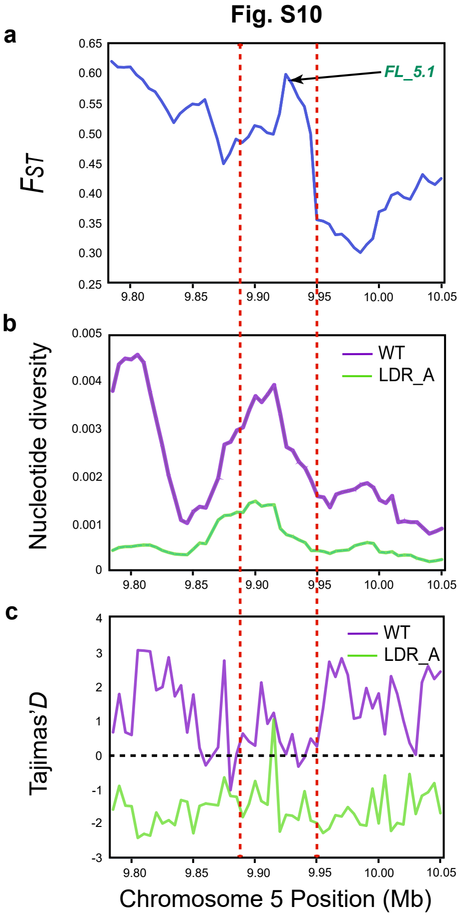

Fig. S11

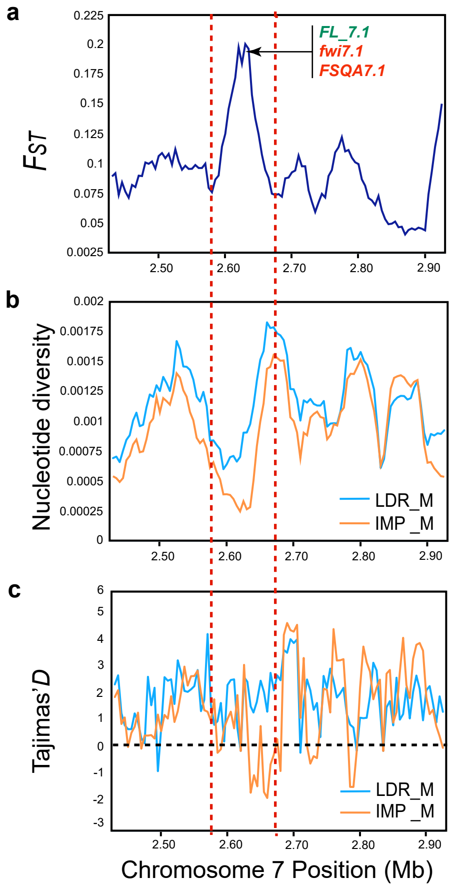

Fig. S4 to **Fig. S11** Potential fruit size selective sweeps.

a, F_{ST} values in the fruit size selective sweep region. **b**, The nucleotide diversity (π) values between different sub-groups in the fruit size selective sweep region. **c**, Tajimas' *D* values between different sub-groups in the fruit size selective sweep region. The two red dashed lines defined the regions contained fruit size GWAS signals (FL, green; FD, dark pink) or previously reported QTLs (red). The black dashed lines indicate the threshold of Tajimas' *D*.

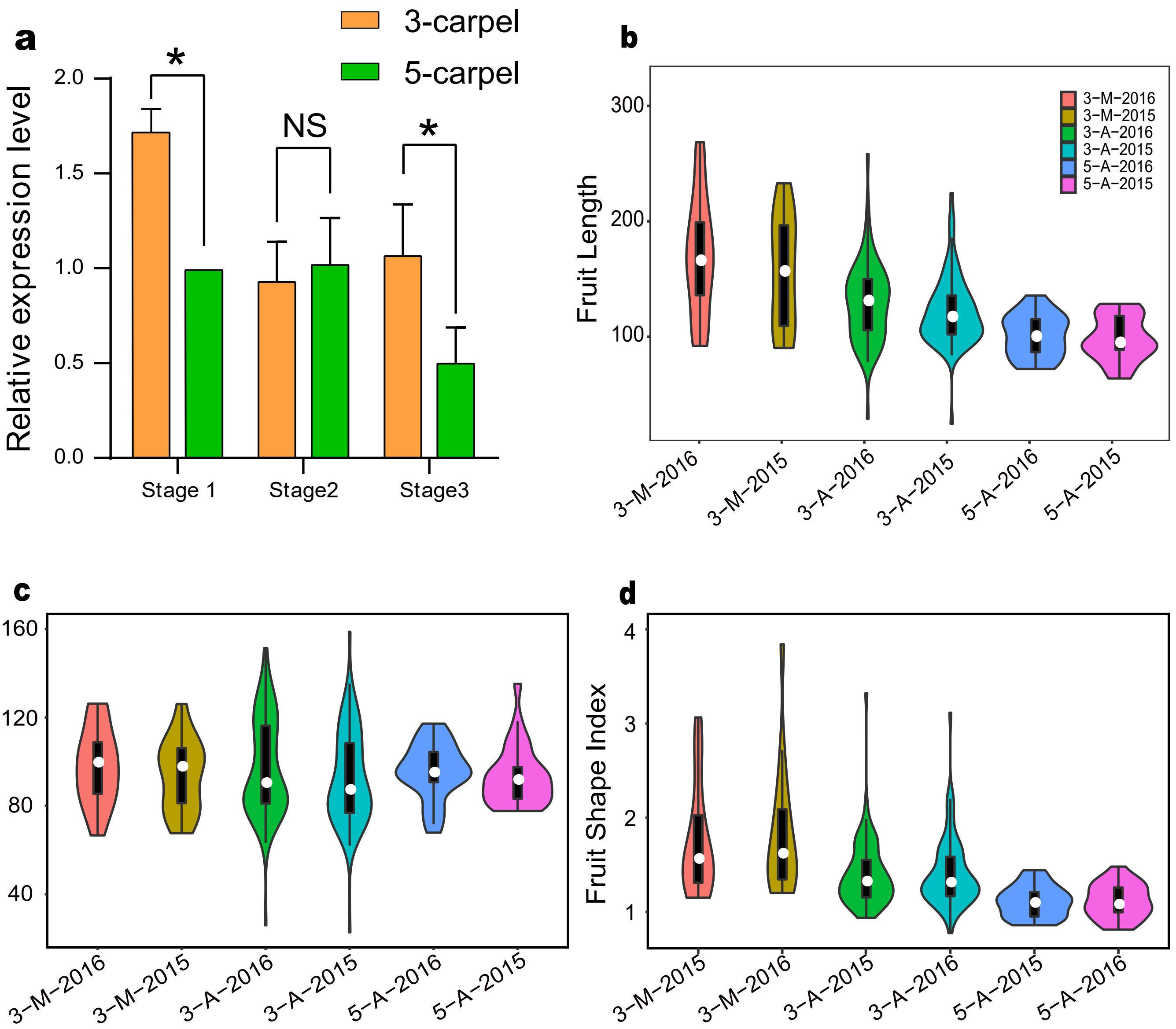

Fig. S12 *CmCLV3* expression pattern in different carpel number melon accessions during melon ovary development and the variations of fruit length, fruit diameter, fruit shape index and sex expression among different carpel number melon accessions.

a, *CmCLV3* expression pattern in different carpel number melon accessions during melon ovary development. The x axis listed different sampled collection stages. Stage 1: 2 mm ovary length; Stage 2: 4-5 mm ovary length; Stage 3: ovary at anthesis. The y axis quantifies the Mean ± Standard Deviation (SD) of the relative expression level. The black asterisk indicated the significant difference and the "NS" indicated "no significant difference" at $p<0.05$ level. **b**, Fruit length variations with sex expression among different carpel number melon accessions. **c**, Fruit diameter variations with sex expression among different carpel number melon accessions. **d**, Fruit shape index variations with sex expression among different carpel number melon accessions. Aandromonoecious (A); monoecious (M); 3 and 5 indicate the 3-carpel (3) and 5-carpel (5) are marked in Fig. (**b**) to (**d**). 2015 and 2016 stand for the phenotype data collected in the year of 2015 and 2016, respectively.

Fig. S13

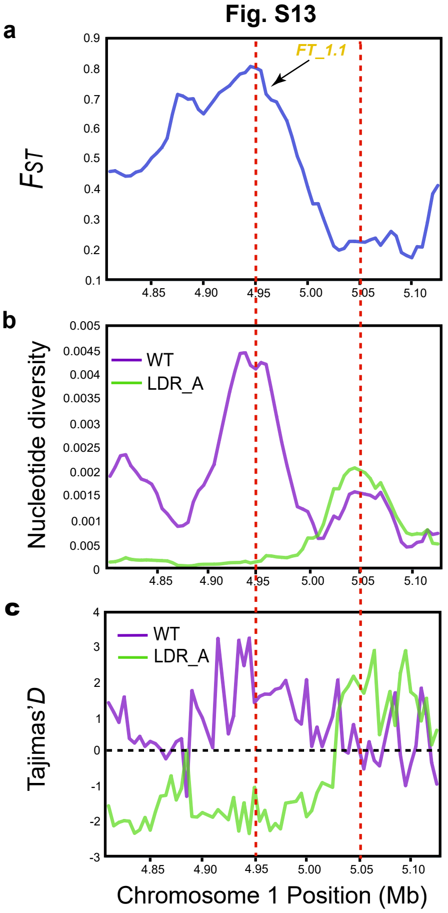

Fig. S14

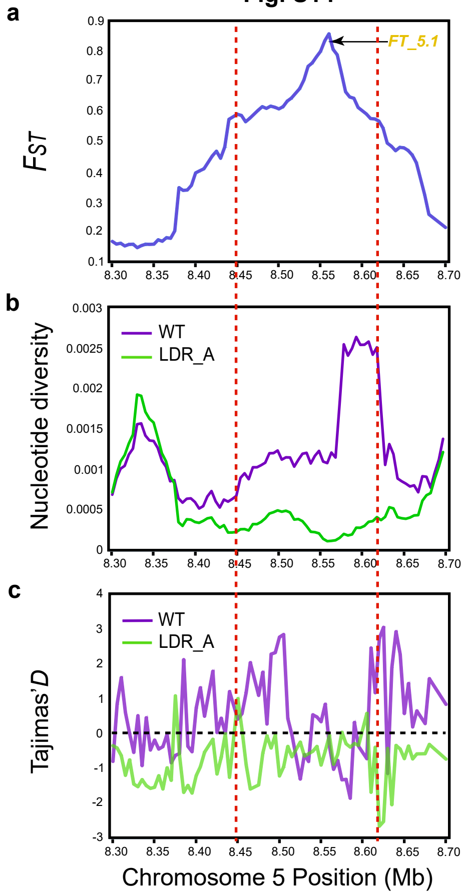

Fig. S15

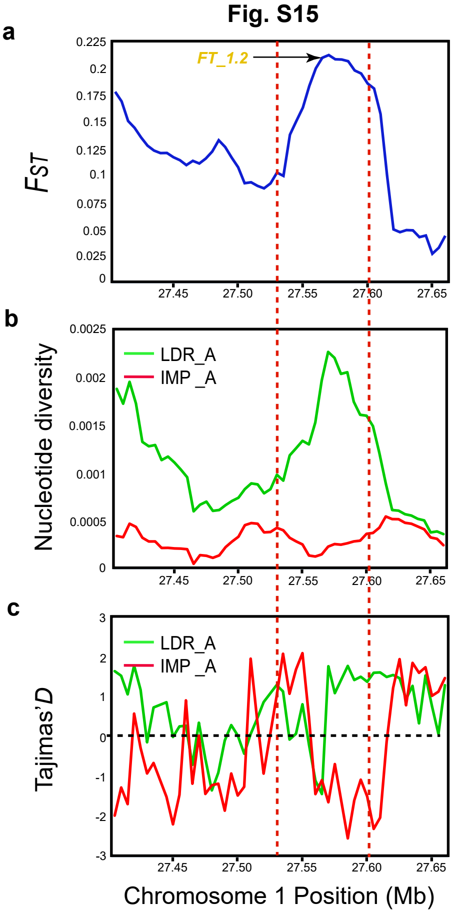

Fig. S16

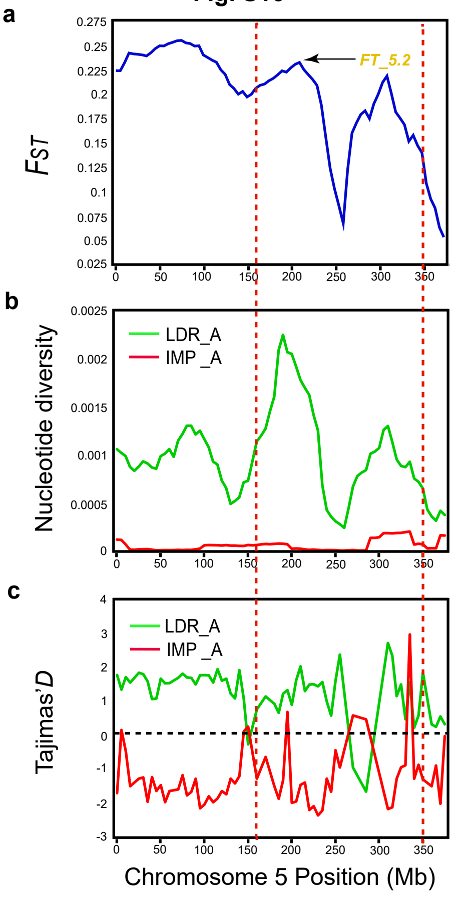

Fig. S17

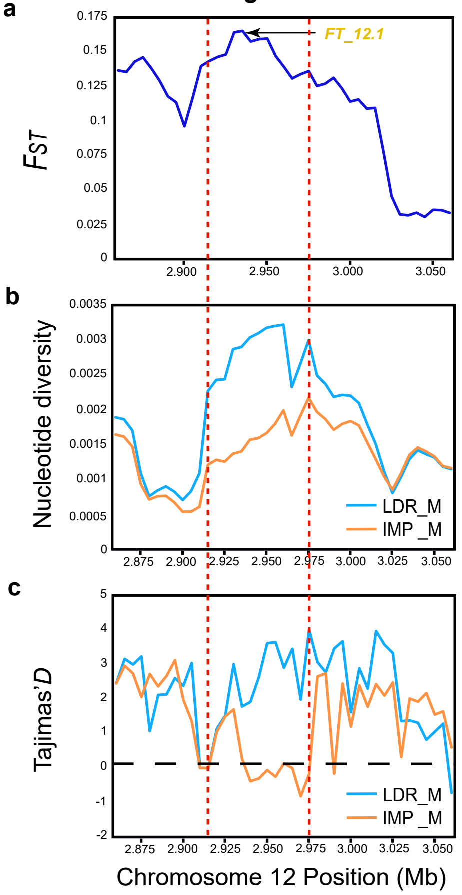

Fig. S18

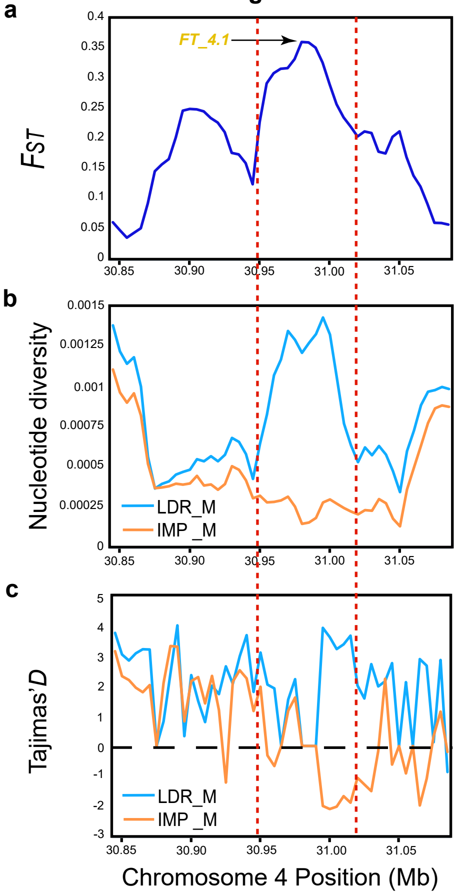

Fig. S19

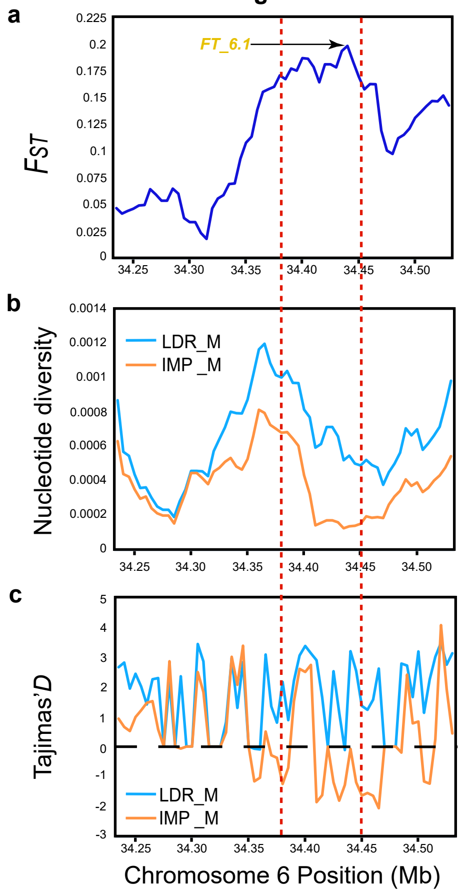

Fig. S13 to **Fig. S19** Potential flesh thickness selective sweeps.

a, F_{ST} values in flesh thickness selective sweep region. **b**, The nucleotide diversity (π) values between different sub-groups in flesh thickness selective sweep region. c, Tajimas' *D* values between different sub-groups in flesh thickness selective sweep region. The two red dashed lines defined the regions contained fruit size GWAS signals (FT, yellow). The black dashed lines indicate the threshold of Tajimas' D.

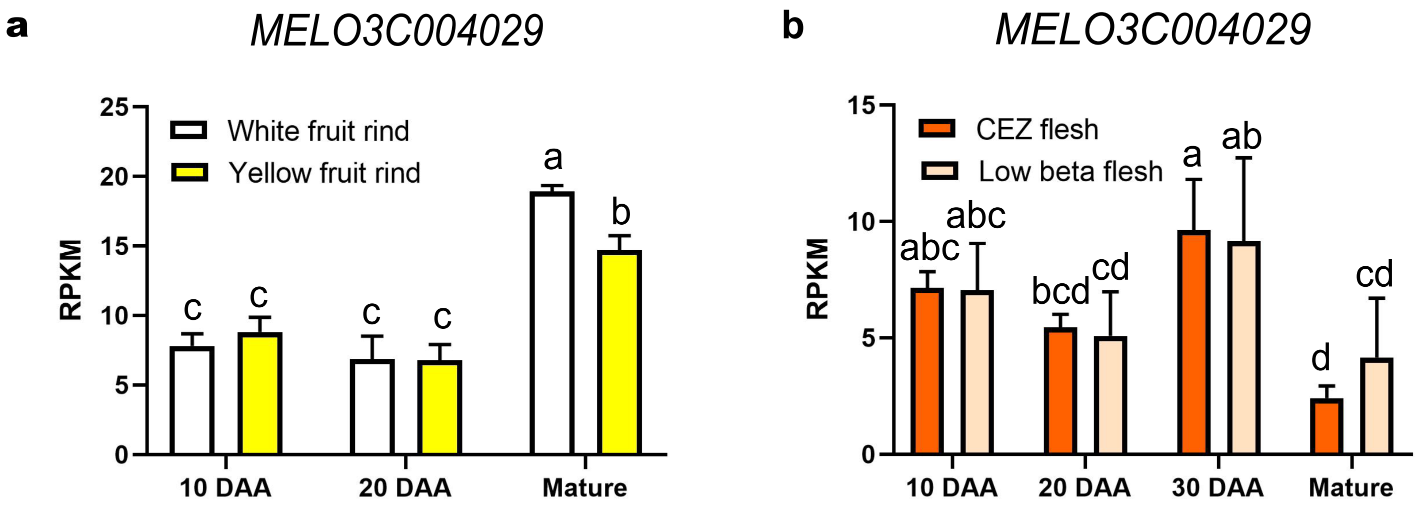

Fig. S20 Expression pattern analysis of *MELO3C004029* with published RNA-seq data during melon fruit development.

a, *MELO3C004029* expression pattern in fruit rind during melon fruit development stages with the BioProject number of PRJNA314069. **b**, *MELO3C004029* expression pattern in fruit rind during melon fruit development stages with the BioProject number of PRJNA288543. Days after anthesis (DAA) are marked under the x axis. The y axis quantifies the Mean ± Standard Deviation (SD) of the RPKM value (n = 3). The significant difference was indicated with “a”, “b”, “c” and “d” at $p<0.05$ level.

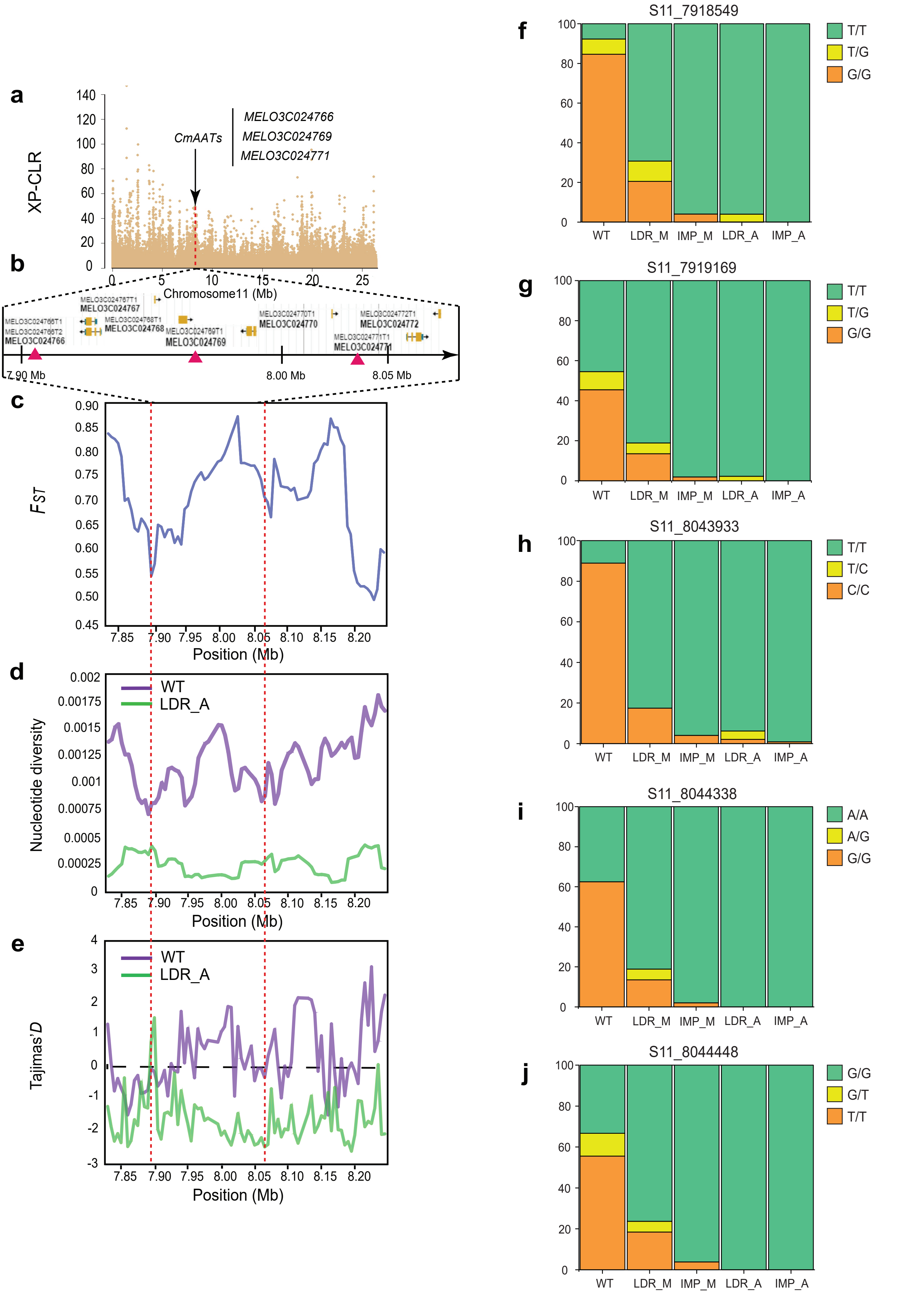

Fig. S21 The putative aroma selective sweep conferred *CmAATs*.

a, XP-CLR analysis on chromosome 11. The red dashed lines and the black arrow indicated the location of selective sweeps contained the *CmAATs*. Three *CmAATs* (*MELO3C024766*, *MELO3C024769* and *MELO3C024771*) were marked with black. **b**, candidate genes in this selective sweep. The red triangles indicated the three *CmAATs*. **c**, F_{ST} values in the selective sweep region contained *CmAATs*. **d**, The nucleotide diversity (π) values between ssp. *agrestis* wild type and landrace sub-groups in the selective sweep region contained *CmAATs*. **e**, Tajimas' *D* values between ssp. *agrestis* wild type and landrace sub-groups in the selective sweep region contained *CmAATs*. The two red dashed lines defined the regions contained *CmAATs*. The black dashed lines indicate the threshold of Tajimas' *D*. **f** to **j**, The genotype variations of SNP (*S11_7918549*, *S11_7919169*, *S11_8043933*, *S11_8044338*, and *S11_8044448*) frequencies among the melon accessions in the five sub-groups, respectively. the x axis quantifies subgroups membership, and the y axis lists the percentage of genotype frequency.

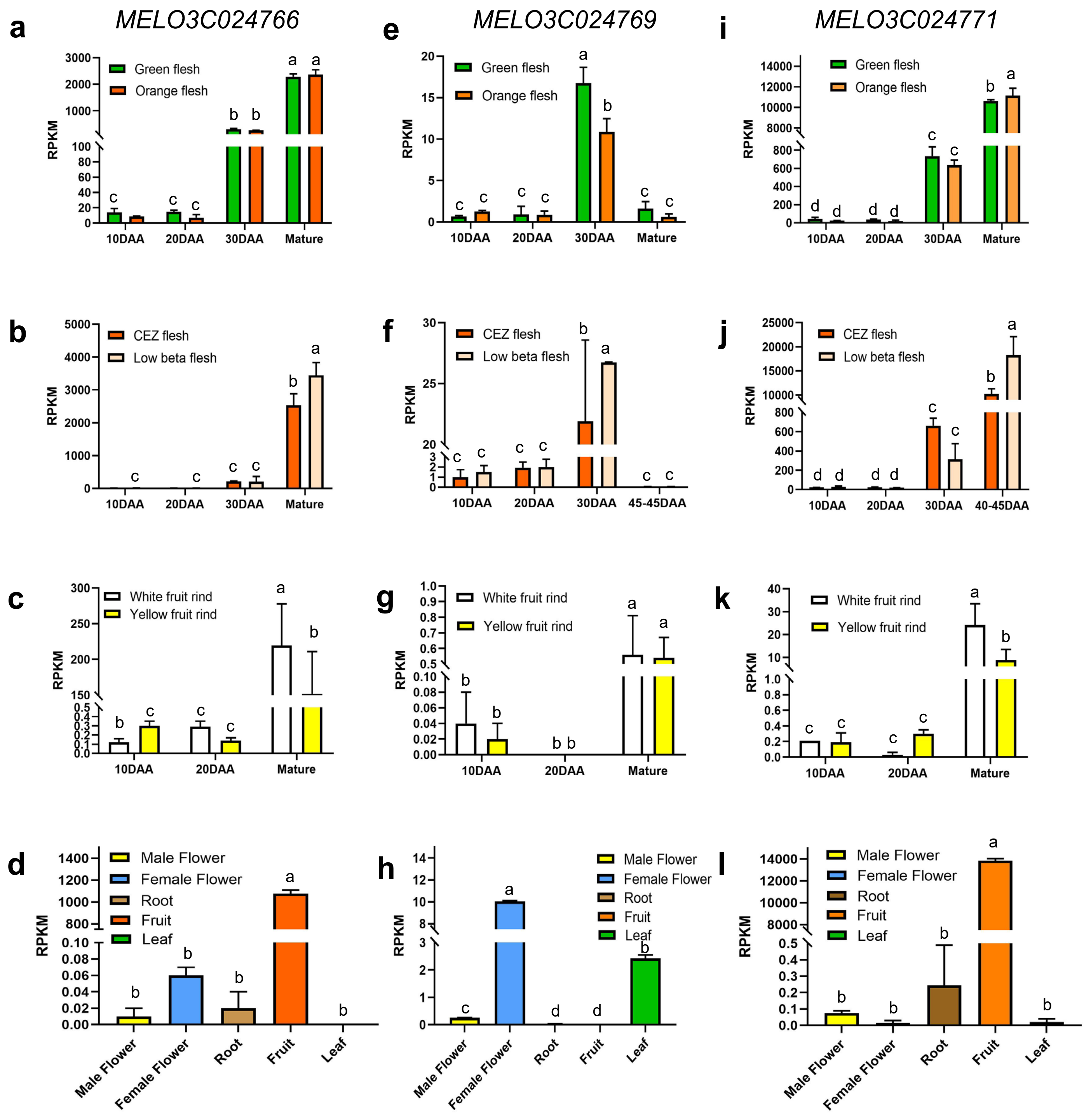

Fig. 22 Expression pattern analysis of *CmAATs* with published RNA-seq data.

a to **d**, The expression analysis of *MELO3C024766* with the BioProject of PRJNA286120, PRJNA288543, PRJNA314069 and PRJNA383830. **e** to **h**, The expression analysis of *MELO3C024769* with the BioProject of PRJNA286120, PRJNA288543, PRJNA314069 and PRJNA383830. **i** to **l**, The expression analysis of of *MELO3C024771* with the BioProject of PRJNA286120, PRJNA288543, PRJNA314069 and PRJNA383830. Days after anthesis (DAA) or different tissues are marked under the x axis, the y axis quantifies the Mean ± Standard Deviation (SD) of the RPKM value (n = 3). The significant difference was indicated with “a”, “b”, “c” and “d” at p<0.05 level.

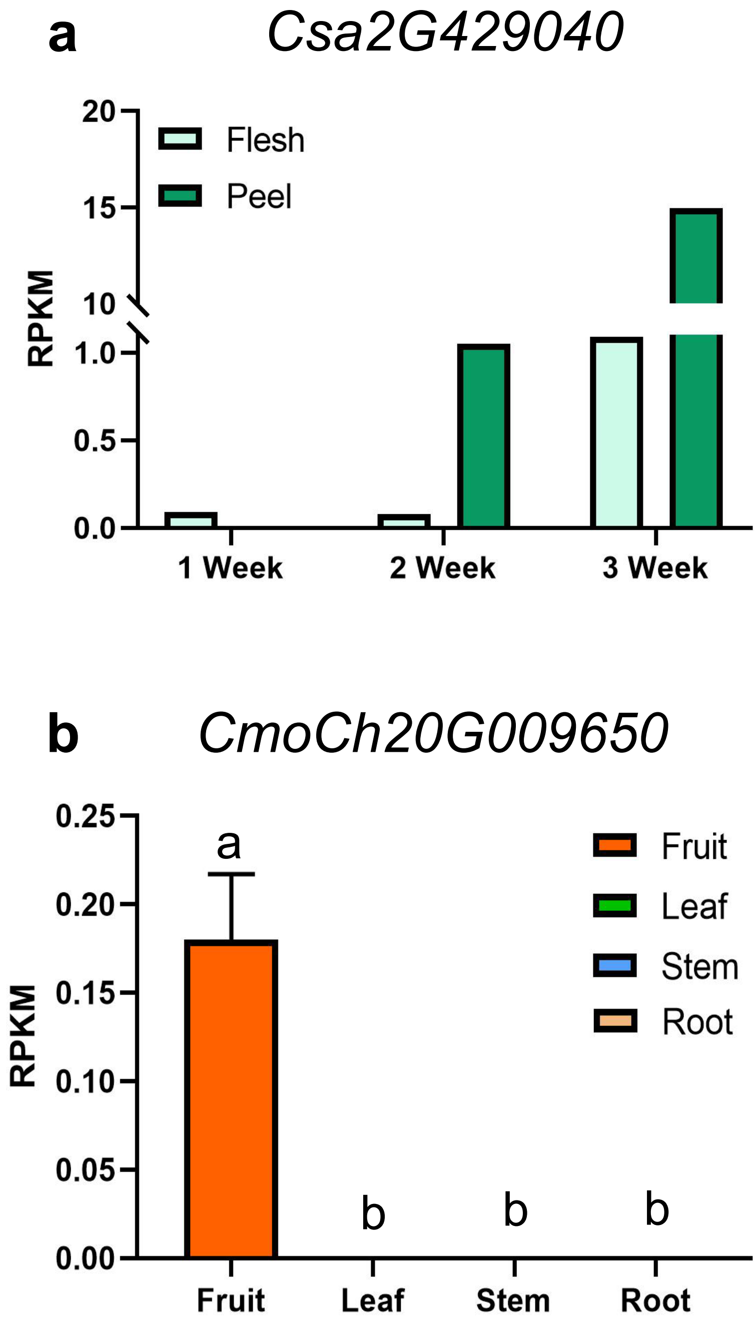

Fig. 23 Expression pattern analysis of *CmAATs* with published RNA-seq data in cucumber and *Cucurbita moschata* (*Rifu*).

a, The expression pattern analysis of *Csa2G429040* in cucumber with the BioProject of PRJNA312872. **b**, The expression pattern analysis among different tissues of *CmoCh20G009650* in *Cucurbita moschata* (*Rifu*) with the BioProject of PRJNA385310. Different sampled stages or tissues are marked under the x axis. The y axis quantifies the Mean ± Standard Deviation (SD) of the RPKM value (n = 3, except Fig. 23a). The significant difference was indicated with “a”, “b”, “c” and “d” at p<0.05 level (except Fig. 23a).
